# Inhibition of the membrane repair protein annexin-A2 prevents tumor invasion and metastasis

**DOI:** 10.1007/s00018-023-05049-3

**Published:** 2023-12-13

**Authors:** C. Gounou, L. Rouyer, G. Siegfried, E. Harté, F. Bouvet, L. d’Agata, E. Darbo, M. Lefeuvre, M. A. Derieppe, L. Bouton, M. Mélane, D. Chapeau, J. Martineau, V. Prouzet-Mauleon, S. Tan, W. Souleyreau, F. Saltel, F. Argoul, A. M. Khatib, A. R. Brisson, R. Iggo, A. Bouter

**Affiliations:** 1 CNRS, Bordeaux INP, CBMN, UMR 5248, University of Bordeaux, Bât. B14, Allée Geoffroy Saint Hilaire, 33600 Pessac, France; 2 INSERM, BRIC, U 1312, University of Bordeaux, 33000 Bordeaux, France; 3 CNRS, LOMA, UMR 5798, University of Bordeaux, 33400 Talence, France; 4 Animalerie Mutualisée, Service Commun des Animaleries, University of Bordeaux, 33000 Bordeaux, France; 5 CRISPRedit, TBMcore, UAR CNRS 3427, Inserm US 005, University of Bordeaux, Bordeaux, France; 6 XenoFish, B2 Ouest, Allée Geoffroy St Hilaire CS50023, 33615 Pessac, France; 7 Bergonié Institute, Bordeaux, France

**Keywords:** Annexins, Invasion, Metastasis, Tumor progression, S100 proteins, MDA-MB-231, AsPC-1

## Abstract

Cancer cells are exposed to major compressive and shearing forces during invasion and metastasis, leading to extensive plasma membrane damage. To survive this mechanical stress, they need to repair membrane injury efficiently. Targeting the membrane repair machinery is thus potentially a new way to prevent invasion and metastasis. We show here that annexin-A2 (ANXA2) is required for membrane repair in invasive breast and pancreatic cancer cells. Mechanistically, we show by fluorescence and electron microscopy that cells fail to reseal shear-stress damaged membrane when ANXA2 is silenced or the protein is inhibited with neutralizing antibody. Silencing of ANXA2 has no effect on proliferation in vitro, and may even accelerate migration in wound healing assays, but reduces tumor cell dissemination in both mice and zebrafish. We expect that inhibiting membrane repair will be particularly effective in aggressive, poor prognosis tumors because they rely on the membrane repair machinery to survive membrane damage during tumor invasion and metastasis. This could be achieved either with anti-ANXA2 antibodies, which have been shown to inhibit metastasis of breast and pancreatic cancer cells, or with small molecule drugs.

**Supplementary Information:**

The online version contains supplementary material available at 10.1007/s00018-023-05049-3.

## Introduction

In cells exposed to mechanical stress, plasma membrane disruption is a physiological event that occurs frequently, especially in muscle, epithelial or endothelial cells, submitted respectively to muscle contraction/stretching, and fluid or hemodynamic shear stress [1]. These cells possess a membrane repair machinery enabling to reseal injuries on a minute scale [1]. The absence of membrane repair leads to cell death and may contribute to the development of degenerative diseases such as muscular dystrophies [2]. Influx of Ca^2+^ from the extracellular (mM) to the intracellular (µM) milieu is the main trigger of membrane repair, which mainly relies on proteins that bind to membranes in a Ca^2+^-dependent manner, such as dysferlin, AHNAK, members of the S100 family, ESCRT machinery or annexins [3–7]. Twelve members, named ANXA1 to ANXA13 (the number 12 is not assigned), compose the annexin family in mammals [8]. They are cytosolic proteins that share the property of binding to membranes exposing negatively-charged phospholipids, when Ca^2+^ concentration rises. Annexins are involved in membrane repair at different stages. ANXA1 and ANXA2 may trigger the fusion of intracellular vesicles and the recruitment to the disruption site of the new-formed lipid patch, which is responsible for membrane resealing [3]. Triggered by the increase in Ca^2+^ concentration, members of the S100 family, notably S100A10 and S100A11, form complexes with ANXA1 and ANXA2 that participate to membrane repair [9–12]. ANXA5 self-assembles around the disruption site to strengthen the membrane, preventing the expansion of the tear, and thus facilitating the repair process [4, 13, 14]. ANXA4 and ANXA6 are also essential for membrane repair by remodeling the damaged plasma membrane, though their specific role remains to be clarified [5, 15].

Cancer metastasis results from a cascade of events that cause the disease to spread through blood or lymph, from the primary tumor to other organs. All along these processes, cancer cells are exposed to permanent mechanical stresses, which change in geometry, scale, and strength, depending on the transformation they are involved in [16]. From volume compression in the primary tumor, mechanical constraints evolve towards hemodynamic and mechanical shear stress when metastatic cells travel the bloodstream, and finally towards adhesive stretch and tight constriction, when they reach very narrow vessels and process to extravasation throughout endothelium, respectively [17, 18]. These mechanical stresses are susceptible to create plasma membrane disruption in cancer cells, which account for an efficient membrane repair machinery to cope with such damages [16, 19]. Although very powerful, the membrane repair machinery may be fragile and could be disturbed, constituting the Achilles' heel of cancer cells. Verifying such a hypothesis would open the way to the development of new therapeutic strategies to hamper tumor invasion and annihilate metastasis. While few studies have associated membrane repair and cancer, many publications have fortuitously reported a positive correlation between key players of membrane repair, such as annexins or S100 proteins, and tumor invasion [9, 11, 20–23]. In addition, our experimental data provided a proof of principle in cellulo [24], by showing that the migration of cancer cells on fibrillar collagen induces membrane damages, whose resealing involve annexins that are highly expressed in invasive cancer cells [24]. When annexins are silenced, cancer cell migration on fibrillar collagen leads to cell death due to a defect in membrane repair [24].

The main objective of the current study was to assess if the inhibition of a key membrane repair protein in cancer cells may affect in vivo tumoral progression. ANXA2 was expected to provide a relevant target, since it promotes cancer progression in various cancer types [25], including breast cancer [26], pancreatic cancer [27], or glioblastoma [28]. In addition, ANXA2 plays a crucial role in membrane repair of many cell types, including cancer cells [9, 29–32]. The aggressive, highly invasive triple-negative breast cancer cell line, MDA-MB-231, and the invasive pancreatic cancer cell line, AsPC-1, were used in this study. We show that the genetic inhibition of ANXA2 prevents tumor invasion and metastasis processes when these cells are injected in mice or zebrafish. We demonstrate that the deficiency in ANXA2 inhibits response of cancer cells to shear stress, which leads to cell death due to the absence of membrane repair. Finally, we show that a monoclonal anti-ANXA2 antibody interferes with the function of ANXA2 in membrane repair of cancer cells, suggesting that it may constitute a relevant tool to inhibit tumor invasion and metastasis.

## Materials and methods

### Ethical issues

Accommodations and experiments were performed in the animal facility (“Service des animaleries”) of the University of Bordeaux. Female RAGγ2C − / − (RAG within the manuscript) or NOD SCID mice were housed and treated in the animal facility. RAG mice were obtained from the animal facility of the University of Bordeaux and NOD SCID mice were purchased from Charles River Laboratories (Wilmington, MA, USA). All animal procedures have been done according to the institutional guidelines and approved by the local ethics committee (agreement number: APAFIS#28418-2020110415441210 v7). Adult zebrafishes were produced in our facilities in accordance with the French Directive (Ministère de l’Agriculture et de l’Alimentation) under permit number A33-063-935. All the procedures were conducted in compliance with the European Communities Council Directive (2010/63/EU).

### Cell culture

Cell culture media and reagents were from ThermoFisher Scientific (Waltham, MA, USA) except when otherwise stated. MDA-MB-231 human breast tumor cells expressing tdTomato and luciferase proteins were established by lentiviral transduction as previously described [33] using the pDRM18 LTN plasmid (Addgene number 174721). MDA-MB-231 and MCF-7 cells were cultured in Dulbecco modified Eagle's minimal essential medium (DMEM) containing 4 mM Glutamax© and supplemented with 10% fetal bovine serum and penicillin / streptomycin (100 U/mL and 100 μg/mL). AsPC-1 human pancreatic tumor cells were modified to express tdTomato protein. Lentiviral vector containing tdTomato gene (pRRLsin-MND-hPGK-tdTomato-WPRE) under the control of the myeloproliferative sarcoma virus enhancer was constructed and produced by the Vect’UB facility of the University of Bordeaux. AsPC-1 were transduced with lentiviral particles at multiplicity of infection (MOI) of 5 for 72 h and selected by cell sorting as previously described [34]. Cells were cultured in RPMI-1640 medium containing 4 mM Glutamax© and supplemented with 10% fetal bovine serum and penicillin / streptomycin (100 U/mL and 100 μg/mL) at 37 °C in a 5% CO_2_ humidified incubator.

### Generation of ANXA2 knock-down or knock-out cells

Complementary strategies were used to generated ANXA2-deficient cells. Either MDA-MB-231 and AsPC-1 were transduced with ANXA2-targetting shRNAs, or an ANXA2-knock-out MDA-MB-231 cell line was established using a CRISPR-Cas9 approach. For the knockout cell line, two gRNA targeting ANXA2 DNA sequences (gRNA1 5ʹ GGTCCTTCTCTGGTAGGCGA-3ʹ or gRNA2 5ʹ-TCTCTGTGCATTGCTGCGGT-3ʹ) located in exon 3 were designed using CRISPOR algorithm (crispor.tefor.net [35]). Alt-R®-crRNA corresponding to target sequence was purchased from Integrated DNA Technologies (IDT) as well as human crRNA negative control. They were both resuspended to 100 µM in TE buffer and then equally mixed with 100 µM Alt-R®-tracrRNA (IDT), annealed by heating for 5 min at 95 °C and cooled to room temperature (RT). This dual gRNA was mixed with 5 µg of Alt-R® S.p-Cas9HIFIv3 (IDT) with a 1.2 ratio of gRNA/Cas9. After 10 min at RT, 2 × 10^5^ MDA-MB-231 cells resuspended in Lonza SE solution were added to the CRISPR mix. Program CH-125 of the 4D-Nucleofector® (Lonza) was applied. Two or three days after transfection, cells were trypsinized and half of them were cultured while the other half was pelleted, lysed and used as PCR template using Phire Tissue Direct PCR Master Mix (ThermoFisher Scientific). PCR amplification of the targeted area was done following supplier instructions with primers 5ʹ-TGGGTAGAGGATGCTGACGA-3ʹ and 5ʹ-CAGAAGCTCTCCCTCCAGGT-3ʹ. Sequencing of PCR products was done by Eurofins Genomics and Sanger data were analyzed to quantify Indels reflecting gene KO with DECODR algorithm (decodr.com [36]) or ICE algorithm (ice.synthego.com [37]). To avoid clonal bias, experiments were performed with the pool of transfected cells rather than cellular clone selected after cell dilution. Regarding the shRNA strategy, the following sequences, which were cloned into the pLKO.1 puro-vector (MISSION® shRNA plasmids, Sigma-Aldrich, Saint-Louis, MO, USA), were used: ANXA2-targetting shRNA: 5ʹ-CCGGCGGGATGCTTTGAACATTGAACTCGAGTTCAATGTTCAAAGCATCCCGTTTTTG-3ʹ; control shRNA: 5′-CCTAAGGTTAAGTCGCCCT-CGCTCGAGCGAGGGCGACTTAACCTTAGG-3′. Lentiviral-based particles containing shRNA were produced by the VectʹUB platform, (INSERM US 005—CNRS UMS 3427-TBM-Core, Université de Bordeaux, France) by transient transfection of 293T cells. 2 × 10^5^ cells were cultured in a 30 mm Petri Dish for 24 h and transduction was carried out by adding concentrated lentiviral particles to the cells at MOI of 10 in 2 mL Opti-MEM® for 24 h. Transduced cells were cultured for 24 h in growth medium and then selected in selection medium composed by 2 µg/mL puromycin in DMEM or RPMI-1640 for 48 h. Cells were passed and subsequently cultured in 25 cm^2^ cell culture flask in selection medium. At each passage, a fraction of cells was used for preparing protein extracts for western blot analysis of the expression of endogenous ANXA2.

### Western blot

2 × 10^6^ cells were trypsinized, pelleted and re-suspended in 300 µL of D-PBS supplemented with 1 mM EGTA. Protein extracts were obtained by sonicating ice-cold cell suspension with a Branson digital sonifier (amplitude 20%, duration 2 min, interval 5 s and pulse 5 s). Two successive centrifugations at 13,000*g* for 1 min allowed to remove cell debris. 10 µg protein extracts, together with the Precision Plus Protein Dual Color Standards (Bio-Rad, Hercules, CA, USA) were separated on a 10% SDS-PAGE. Semi-dry electrophoretic transfer (Bio-Rad) onto PVDF membrane was performed for 1 h at 100 V. The cellular content in ANXA1 (37 kDa), ANXA2 (36 kDa), ANXA4 (35 kDa), ANXA5 (35 kDa), ANXA6 (68 kDa), and actin (42 kDa) or GAPDH (37 kDa) was detected with rabbit anti-ANXA1 polyclonal antibody (PA1006, BosterBio, Pleasanton, CA, USA), mouse anti-ANXA2 monoclonal antibody (3E8-B6, Sigma-Aldrich), mouse anti-ANXA4 monoclonal antibody (SAB4200121, Sigma-Aldrich), mouse anti-ANXA5 monoclonal antibody (AN5, Sigma-Aldrich), mouse anti-ANXA6 monoclonal antibody (sc-271859, Santa cruz Biotechnology, Dallas, USA), rabbit anti-actin polyclonal antibody (A2066, Sigma-Aldrich), and rabbit anti-GAPDH polyclonal antibody (G9545, Sigma-Aldrich), respectively. Except for anti-actin and anti-GAPDH antibodies (1:5000), all primary antibodies were used at 1:1000 dilution in saturation solution composed by Tris buffer saline (10 mM Tris, 150 mM NaCl, pH 8.0) supplemented with 0.1% Tween20 and 5% nonfat dry milk. Revelation was performed using secondary antibodies conjugated to horse-radish peroxidase (GE-Healthcare, Chicago, USA) diluted 1:2000 in saturation solution and Opti-4CN™ colorimetric kit (Bio-Rad). ImageJ software was used to measure the relative intensity of protein bands.

### Membrane rupture and repair assay

Membrane repair assay was performed as previously described [24]. Cells were irradiated at 820 nm with a tunable pulsed depletion laser Mai Tai HP (Spectra-Physics, Irvine, USA) of an upright two-photon confocal scanning microscope (TCS SP5, Leica, Wetzlar, Germany) equipped with an HCX PL APO CS 63.0 × 1.40 oil-objective lens. Irradiation consisted of 1 scan (1.6 s) of a 1 µm × 1 µm area with a power of 110 (± 5) mW. 512 × 512 images were acquired at 1.6 s intervals with pinhole set to 1 Airy unit. FM1-43 was excited by the 488-nm laser line (intensity set at 20% of maximal power) and fluorescence emission was measured between 520 and 650 nm. For quantitative analysis, the fluorescence intensity was integrated over the whole cell surface and corrected for the fluorescence value recorded before irradiation, using ImageJ software.

### Traffic of ANXA2 in laser-injured cells

For subcellular localization of endogenous ANXA2 in damaged cells, cells were cultured in 35-mm glass bottom dishes equipped with a square patterned coverslip (MatTek, Ashland, USA) and membrane rupture was performed according to the protocol described above, but in the absence of FM1-43 to avoid fluorescence cross-talk. After laser irradiation, cells were fixed in 1% glutaraldehyde and permeabilized in 0.1% TritonX100 diluted in DPBS + Ca^2+^. All subsequent steps (saturation, antibody incubation and washes) were performed using 2% BSA in DPBS + Ca^2+^ solution. Primary mouse anti-ANXA2 monoclonal antibody (1:100, 3E8-B6, Sigma-Aldrich) and secondary Alexa Fluor 488-coupled antimouse IgG goat antibody (1:1000, ThermoFisher Scientific) were successively incubated with cells for 1 h at 37 °C. Finally, cells were washed in DPBS + Ca^2+^ and nuclear counterstaining was performed with DAPI (Sigma-Aldrich). For each condition, about 30 cells from three independent experiments were analyzed. For the subcellular trafficking analysis of ANXA2-GFP, cells cultured in 35-mm glass bottom dishes (MatTek, Ashland, USA) were transfected with the pA2-GFP plasmid [38], as previously described [15]. Membrane damage was performed by laser ablation as described above, without FM1-43. At least three independent experiments were performed and each experiment included the analysis of at least five damaged cells.

### 2-D migration assay

6-Well clear plates (Corning, NY, USA) were coated with gelatin 0.5 mg/mL (Sigma-Aldrich) and incubated for 20 min at 37 °C. Gelatin was crosslinked by incubation with glutaraldehyde 0.5% (Sigma-Aldrich) for 40 min at 37 °C. A 2-chamber culture-insert (Ibidi, Gräfelfing, Germany) was placed in the center of each well. 3 × 10^4^ cells in 1 mL of complete growth medium was added in the well and each culture-insert chamber was filled with 2 × 10^3^ cells in 70 µL growth medium and incubated at 37 °C, 5% CO_2_. The culture-insert was then carefully removed and 2 ml of complete growth medium were added into the well. Quantitative phase images were acquired with a lens-free microscope (Cytonote, Iprasense, Montpellier, France) in an incubator at 37 °C, 5% CO_2_. The acquisition period was 20 min. Analysis was performed on ImageJ using the MRI Wound Healing Tool.

### Proliferation analysis

6-Well clear plates (Corning) were coated with gelatin as described for migration assay. 1.6 × 10^5^ cells/well were seeded in triplicate in growth medium without red phenol to avoid disturbance of the phase measurement. Quantitative phase images were acquired with a lens-free microscope (Cytonote, Iprasense, Montpellier, France) in an incubator at 37 °C, 5% CO_2_. The acquisition period was 25 min. At least three independent experiments were performed for each cell line. Phase images were analyzed using Trackmates2 on Fiji and MATLAB.

### Fluid shear-stress assay

Cells were collected at 80% confluence after incubation with 0.25% trypsin (Gibco) in 5% CO_2_ at 37 °C and 5 × 10^5^ cells were suspended in 1 mL of growth medium in a Falcon™ polystyrene tubes (Corning). At this stage either Ca^2+^ (Final concentration 2 mM), or 2 mM EGTA, or mouse anti-ANXA2 monoclonal antibody (1:100, 3E8-B6, Sigma-Aldrich) supplemented with Ca^2+^ (Final concentration 2 mM) was added to the medium. The suspension was slowly loaded into a 1 ml syringe (Becton Dickinson, NJ, USA), which was subsequently equipped with a 30G needle (Terumo, Tokyo, Japan). Then, the suspension was gently expelled against the wall of the polystyrene tube at room temperature at a constant flow rate either via an automated NeMESYS syringe pump (200 µL/s, Cetoni GmbH, Korbussen, Germany) or manually. The load/expel cycle was repeated 10 times. 10 min after the treatment, 50 µL of the cell suspension were put into a 96-well plate and 4 µg/mL DAPI was added in order to stain damaged cells. As a control condition, cells in growth medium with Ca^2+^ (Final concentration 2 mM) were incubated with 4 µg/mL DAPI without to be submitted to shear stress (no-stress condition). Cell imaging of tdTomato (all cells) and DAPI (unrepaired cells) was performed as described in the Fluorescence microscopy section. Images were analyzed with ImageJ software.

### 3-D invasion assay

Transwell chambers (8.0 µm pore size, Falcon 353097) were coated with 150 µl of Matrigel (80 µg/ml) or fibrillar collagen (0.5 mg/ml) in a 24-well plate. For each condition, 4 × 10^4^ cells (control or ANXA2-deficient) per transwell were seeded in complete medium with 5% of FBS. As a chemoattractant, 20% FBS complete medium was used in the lower part of the transwell in the 24-well plate. After 24 h, the media was carefully removed, cells were fixed with 4% PFA for 10 min and stained with crystal violet (Sigma-Aldrich) for 20 min at room temperature. Cells in the upper part of the transwell membrane were removed by wiping with a cotton swab. A phase-contrast microscope (Zeiss) was used to image migrated cells in the lower chambers, 10 images per condition and per replicate were acquired. Three independent experiments were performed. Images were analyzed using ImageJ software.

### Fluorescence microscopy

Cell imaging was performed using a conventional fluorescence microscope IX81 (Olympus, Tokyo, Japan) equipped with a UPLFLN20X/0.50/WD2.1mm and a UPLFLN60XO/0.65/0.65–1.25/D0.12 objectives. DAPI and Hoechst were observed using the U-MWU2 cube containing a bandpass excitation filter (330–385 nm), a dichroic mirror (threshold 400 nm) and a long-pass emission filter (threshold 420 nm). GFP and Alexa488 were observed using the U-MINIBA2 cube containing a bandpass excitation filter (470–490 nm), a dichroic mirror (threshold 505 nm), and a bandpass emission filter (510–550 nm). tdTomato was observed using the cube U-MWG2 containing a bandpass excitation filter (510–550 nm), a dichroic mirror (threshold 570 nm) and a long-pass emission filter (threshold 590 nm).

### Transmission electron microscopy

5 × 10^5^ cells submitted or not to shear stress assay were centrifuged at 200*g* for 5 min in a 1.5 mL polypropylene tube. The pellet was incubated with a solution of Karnovsky fixative at 4 °C overnight. The pellet was washed with 0.1 M sodium cacodylate buffer (Agar Scientific, Stansted, UK), then post-fixed for 1 h in 1% osmium tetroxide (Agar Scientific) in sodium cacodylate buffer and then washed three times and finally re-suspended in 20 µL of cacodylate buffer. A 5 µL drop was introduced into the core of a warm (40–50 °C) 8% agarose fluid gel in a 1.5 mL polypropylene tube. Once solidified, a 3 mm^3^ block was cut around the drop. Dehydration process was performed by 3 successive baths of 10 min in 100% ethanol followed by incubation in propylene oxide. The sample was then embedded in Epon-Araldite and ultra-thin sections (65 nm with Leica EM-UC6 ultra-microtome) were stained for 10 min in 5% uranyl acetate and 5 min in lead citrate. The sections were imaged with a FEI CM120 transmission electron microscope at 120 kV, using a Gatan USC1000-SSCCD camera.

### Cancer cell dissemination in mice

Cancer cells expressing the luciferase protein, either wild-type or ANXA2 deficient, were trypsinized and washed with PBS twice. Female RAG or NOD SCID mice (8–12 weeks old) were anaesthetized with 2% isoflurane. 1 × 10^6^ cells in 0.1 ml DMEM medium were injected into the lateral tail vein using a 27 1/2 gauge needle. For bioluminescence imaging, intraperitoneal injection of d-Luciferin (150 mg/kg) was performed. The mice were immediately placed onto a black pad in the Photon Imager™ (BIOSPACE, Paris, France) box and imaged ventrally. Animals were imaged 30 min after cell injection and then once a week for at least 5 weeks. For image acquisition, unit was set to ph/cm^−2^/s^−1^/sr^−1^. To quantify the signal, a region of interest was chosen as a circle of 13 cm^2^ centered either on lungs or the hindquarters of the mouse.

### Cancer cell dissemination in zebrafish

Experiments were performed using the Casper zebrafish stain (ZIRC, Oregon, USA). Adult fish aged 6 months to 2 years were crossed to produce embryos. Embryos were cultured in E3 medium (5 mM NaCl, 0.17 mM KCl, 0.33 mM CaCl_2_, 0.33 mM MgSO_4_) at 28 °C. Dechorionated 2-day post fertilization (dpf) zebrafish embryos were anaesthetized with 0.003% tricaine (Sigma*-*Aldrich) and positioned in 3% methylcellulose on a dish coated with 1% agarose. Cancer cells expressing the tdTomato protein, either wild type or ANXA2 deficient, were treated with versene solution and resuspended in PBS containing 1% phenol red at the density of 4 × 10^7^ cells per mL. The cell suspension was loaded into borosilicate glass capillary needles (Femtotip II, Eppendorf, Hamburg, Germany) and injections were performed using a pump (Femtojet 4i; Eppendorf) and micromanipulator (Phymep, Paris, France). Around 500 cells/embryos were injected above the duct of cuvier in perivitelline space of the embryo. After checking the implantation with mammalian cells, zebrafish embryos were maintained at 35.5 °C. Tumor imaging is done at 3-, 28- and 52-h post injection (hpi).

### Immunohistochemistry

Murine lungs were fixed in PFA 4% for 48 h at 4 °C progressively dehydrated in ethanol, incubated in toluene and embedded in paraffin. Five micrometer sections were stained with hematoxylin/eosin or used for immunostaining. For immunostaining, sections were deparaffinized in toluene, rehydrated gradually in ethanol and rinsed in dH_2_O. Slides were permeabilized 15 min in 0.1% TritonX100 and saturated 1 h in 5% BSA in PBS. Anti-tdTomato primary antibody (1:50, DsRed (E-8), Santa cruz Biotechnology) was dissolved in 1% BSA in PBS and incubated overnight at 4 °C. After washes, slices were incubated with secondary Alexa Fluor 488-coupled antimouse IgG goat antibody (1:500, ThermoFisher Scientific) for 1 h at RT. Hoechst at 2 µg/mL in PBS and incubated 5 min at RT. Slices were mounted in ProLong medium and dry at RT before imaging.

## Results

### ANXA2 is highly expressed in MDA-MB-231 breast and AsPC-1 pancreatic cancer cells

**Fig. 1:**
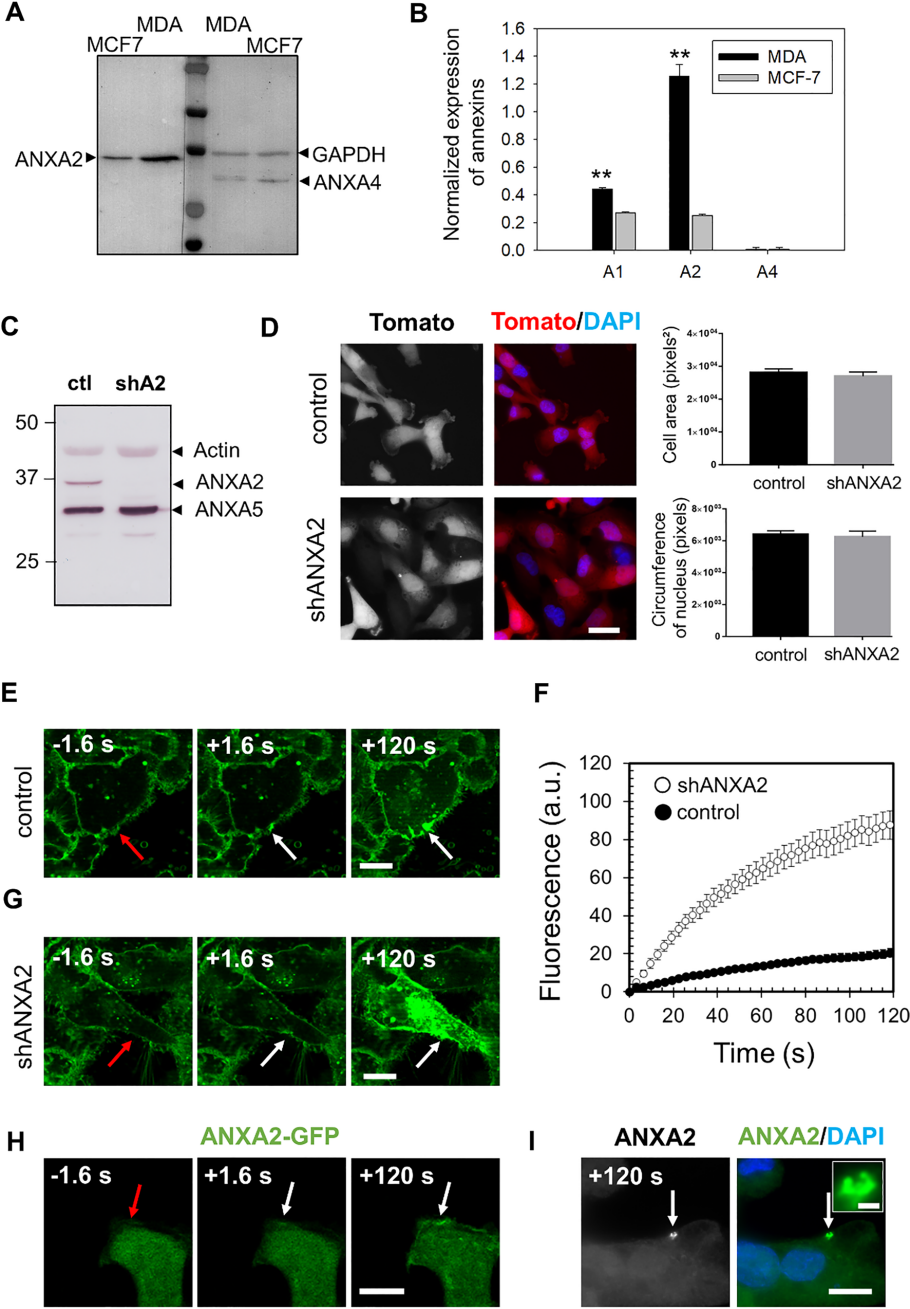
Highly expressed in MDA-MB-231 cells, ANXA2 promotes membrane repair. **A** Representative image of western-blot analysis showing the revelation of ANXA2 in MDA-MB-231 and MCF7 cells, as compared to GAPDH (loading control). **B** The histogram presents mean values (± SEM) of the ratio ANX/GAPDH from five independent experiments, analyzed by the gel analysis plugging of ImageJ. A representative membrane of the detection of ANXA1 is presented in supplementary Fig. S1. Student t test for independent samples. **p < 0.01. **C** ANXA2-deficient MDA-MB-231 cells were generated by shRNA transduction strategy. The cellular content of ANXA2 in MDA-MB-231 cells transduced with lentiviral particles containing shRNA targeting ANXA2 (shA2) or a scrambled shRNA (ctl) was quantified by Western blotting. **D** Control and shANXA2 MDA-MB-231 cells, which expressed constitutively the tdTomato fluorescent protein were imaged by fluorescence microscopy. Right-hand histograms display mean cell area (in pixels^2^) and nuclei circumference (in pixels) measured by the imageJ software using Tomato and DAPI images. The mean values (+ / − SEM) were calculated from at least 30 cells from three independent experiments. No statistical difference (student *t* test) was observed for the two parameters. Scale bar: 20 µm. **E**, **G** Sequences of representative images showing the response of a control (**E**) or shANXA2 (**G**) MDA-MB-231 cell to a membrane damage performed by 110-mW infrared laser irradiation, in the presence of FM1-43 (green). In all figures, the area of membrane irradiation is marked with a red arrow before irradiation and a white arrow after irradiation. Scale bars: 10 μm. **F** Kinetic data represent the FM1 − 43 fluorescence intensity for control (black filled circles) or shANXA2 (empty circles) MDA-MB-231 cells, integrated over whole cell sections, averaged for about 30 cells (+ / − SEM). **H** Recruitment of ANXA2 to the site of membrane injury. MDA-MB-231 cells transfected with the plasmid pA2-GFP were damaged by laser ablation. Red arrow, area before irradiation; white arrow, area after irradiation. I Subcellular localization of endogenous ANXA2 in damaged MDA-MB-231. MDA-MB-231 cells were irradiated with a 110-mW infrared laser (white arrow) in DPBS + Ca^2+^, then fixed and immunostained for ANXA2 and counterstained with DAPI (blue). After laser injury, MDA-MB-231 cells exhibited an accumulation of ANXA2 at the disruption site. The inset displays a magnified image of the disruption site where concentrates ANXA2. Scale bars: 10 µm on the images and 1 µm within the inset

To explore the impact of annexins in breast cancer, we compared the expression of ANXA1, ANXA2 and ANXA4 in MDA-MB-231 and MCF7 cells at the protein (Figs. 1A, B and  S1) and mRNA level (Fig. S2). MDA-MB-231 is a highly invasive, hormone receptor negative breast cancer cell line, whereas MCF7 is an estrogen receptor positive cell line with lower invasiveness properties [39, 40]. We showed that ANXA1 and ANXA2 were expressed at higher levels in MDA-MB-231 cells than in MCF7 cells, as previously observed for ANXA5 and ANXA6 [41]. These results suggest that higher expression of membrane repair genes may contribute to the more invasive phenotype of hormone receptor negative breast tumors. We chose to focus on ANXA2 in the remainder of the study because it showed the highest expression of the annexins tested, as recently reported [42]. Nevertheless, to ensure that results were not specific to the MDA-MB-231 cell line and may concern other invasive cancer cells, AsPC-1 pancreatic cancer cells were studied, showing also a high expression of ANXA2 (Fig. S3A, B).

### Membrane repair in breast and pancreatic cancer cells requires ANXA2

We examined the ability of MDA-MB-231 and AsPC-1 cells to repair membrane damage in vitro after silencing ANXA2 expression either by stable RNA interference, leading to cell lines hereafter named shANXA2 MDA-MB-231 (Fig. 1C–F) and shANXA2 AsPC-1 (Fig. S3C–F), or by CRISPR-mediated deletion of ANXA2 (Fig. S4). We confirmed by Western blotting that ANXA2 protein expression was strongly reduced in shANXA2 cells (Figs. 1C and S3C). Fluorescence microscopy after labelling MDA-MB-231 and AsPC-1 cells with a tdTomato-reporter revealed no significant difference in morphology after silencing ANXA2 expression (Figs. 1D and S3D). To confirm that ANXA2 is required for membrane repair in MDA-MB-231 and AsPC-1 cells, we performed a standard membrane repair assay using laser ablation in the presence of Ca^2+^ and FM1-43 [24, 43]. After laser injury, FM1-43 enters the cytosol where it fluoresces upon incorporation into intracellular membranes. This fluorescence increases until the plasma membrane is resealed. In control MDA-MB-231 or AsPC-1 cells, we observed that FM1-43 entered the cell at the site of membrane irradiation within seconds of laser injury, confirming the presence of membrane rupture (Figs. 1E and S3E, + 1.6 s, arrow). After 120 s, most damaged cells exhibited an increase of intracellular fluorescence limited to the area close to the disruption site (Figs. 1E and S3E, + 120 s, arrow). The kinetics of the change in the fluorescence intensity showed that intracellular fluorescence intensity increased for about 60 s and then reached a plateau (Figs. 1F and S3F, filled circles), indicating rapid resealing of the plasma membrane. In contrast, when shANXA2 (or koANXA2) MDA-MB-231 or shANXA2 AsPC-1 cells were irradiated, they showed a much larger increase in fluorescence intensity (Figs. 1G–F, S3G-F and S4B-C, empty circles), indicating the absence of membrane resealing. Most key membrane repair proteins are rapidly recruited to the site of membrane damage either to participate in the formation of the lipid patch by fusion of intracellular vesicles or for remodeling the damaged plasma membrane [31]. To test whether ANXA2 is recruited to the site of damage, we transfected shANXA2 MDA-MB-231 cells with an ANXA2-GFP vector and analyzed the intracellular trafficking of ANXA2 after membrane injury by laser ablation. We observed that ANXA2-GFP was consistently recruited to the membrane disruption site in a few seconds (Fig. 1H). To rule out a role for GFP in this process, we localized endogenous ANXA2 by immunofluorescence in laser-damaged control cells, as previously described [14, 44]. This confirmed that ANXA2 is recruited to the membrane disruption site (Fig. 1I). We conclude that ANXA2 is required for membrane repair in MDA-MB-231 and AsPC-1 cells.

**Fig. 2:**
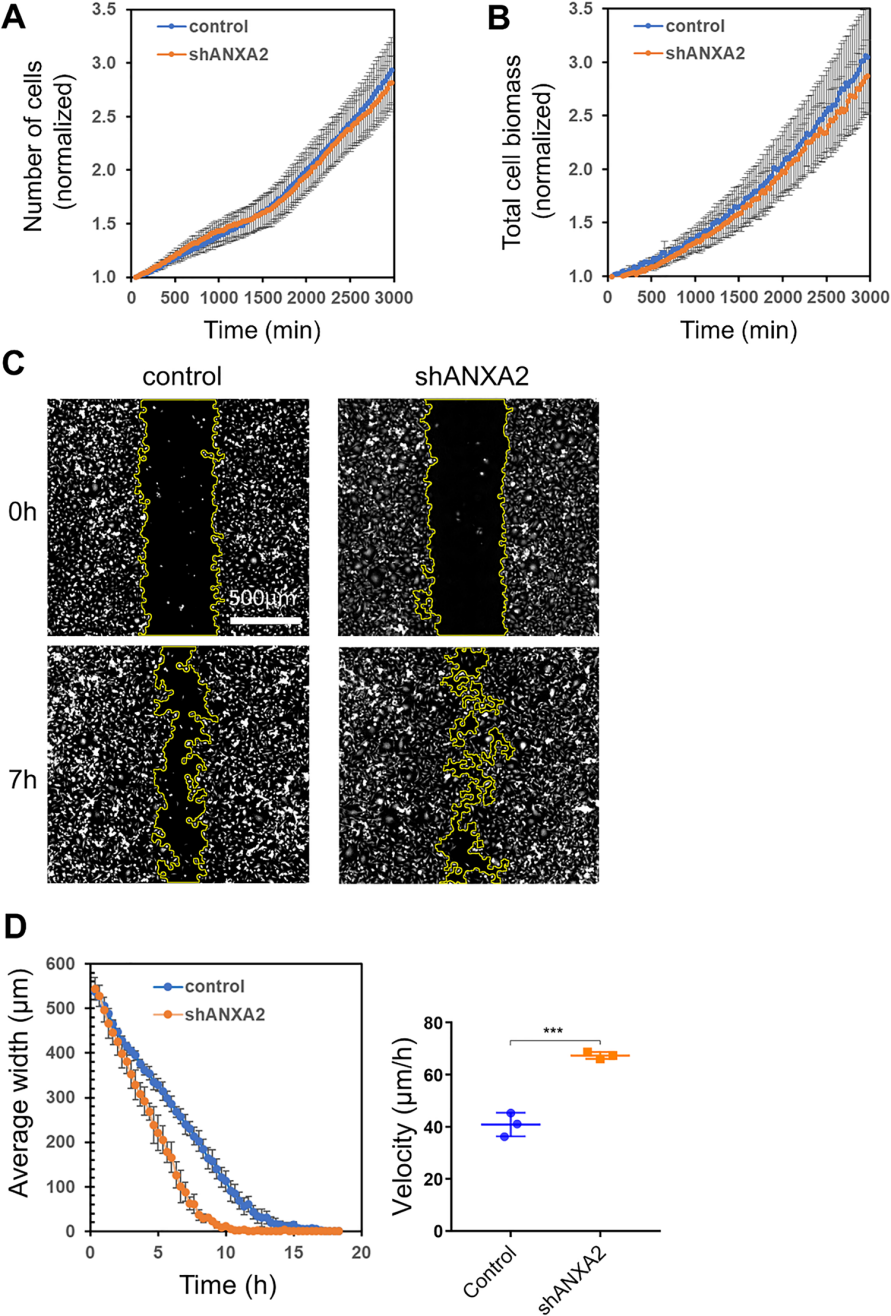
Proliferation and 2D-migration of MDA-MB-231 cells are independent of ANXA2. **A**, **B** Proliferation of control or shANXA2 MDA-MB-231 cells was monitored with a lensfree microscope enabling long-term observation of a large cell population [45]. **A** Averaged number of cells (± SEM) normalized to the number of seeded cells from three independent experiments. **B** Normalized total cell biomass (± SEM), which considers individual number and cell size, from three independent experiments. **C**, **D** In vitro 2D-migration assay was performed on control or shANXA2 MDA-MB-231 cells. Cells were plated into culture-Insert 2-well in 35 mm µ-Dish (Ibidi) coated with 0.5 mg/mL gelatin. Cell progression was monitored with a lensfree microscope, after removing the culture insert. **C** Typical images of control or shANXA2 MDA-MB-231 cells immediately or 7h after insert release are presented. Migration front is drawn in yellow. **D** Mean gap closure kinetics and cell velocity measure (± SEM) from three independent experiments are presented for each cell type. Nonparametric Mann–Whitney test. ***p < 0.001

To investigate whether silencing of ANXA2 expression affects cell growth in vitro, we performed time-lapse imaging with a lens-free microscope. There was no significant difference in the growth rate of ANXA2-deficient MDA-MB-231 or AsPC-1 cells as compared to control cells in vitro, regarding either the number of cells per time unit or the total biomass, which includes in addition the individual cell masses (Figs. 2A, B and S5A, B). To test whether ANXA2 deficiency affects 2D cell migration, a wound healing assay was performed. Using a two-chamber cell-culture insert, a 500 µm-wide cell free gap was created and the ability of control or shANXA2 MDA-MB-231 cells to close the gap was assessed (Fig. 2C). The time required for 80% wound closure was 9.8 ± 1.6 h and 6.7 ± 0.6 h for control and shANXA2 MDA-MB-231 cells, respectively (Fig. 2D). The control and shANXA2 MDA-MB-231 cells migrated at 40.9 ± 4.1 µm/h and 67.4 ± 1.3 µm/h, respectively (Fig. 2D). This slight increase in the speed of wound closure was not observed in koANXA2 MDA-MB-231 (Fig. S5C). In AsPC-1 cells, we observed instead that ANXA2 deficiency decreases the speed of wound closure (Fig. S5D). We conclude that ANXA2 deficiency has no effect on proliferation but may slightly disturbs 2D migration in vitro.

### ANXA2 deficiency impairs membrane repair after shear stress in vitro

**Fig. 3:**
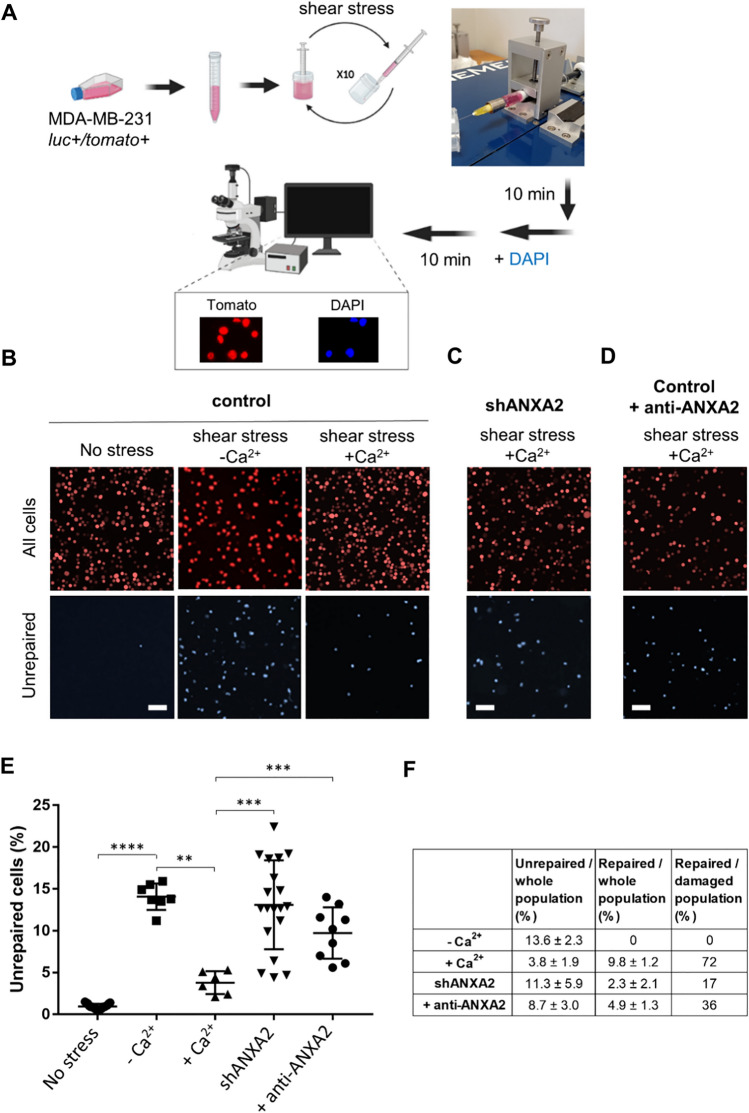
Response of control or shANXA2 MDA-MB-231 cells to membrane injury by shear-stress treatment. **A** Scheme of the protocol with a photography of the device used for the automatized shear-stress treatment. Cells were loaded in a 1 mL syringe with a 30G needle mounted on a fully automated syringe pump system. After 10 passages through the needle, cells were incubated with DAPI, which stained damaged/unrepaired cells, while all cells expressed the fluorescent protein tdTomato. Created using Biorender.com. **B** Representative images of control unstressed MDA-MB-231 (left-hand panels), and stressed cells in the absence (middle panels) or presence (right-hand panels) of 2 mM Ca^2+^. Scale bars: 50 µm. **C** Representative images of shANXA2 MDA-MB-231 submitted to the shear stress assay in the presence of 2 mM Ca^2+^. Scale bar: 50 µm. **D** Representative images of control MDA-MB-231 submitted to shear stress assay in the presence of 2 mM Ca^2+^ and monoclonal anti-ANXA2 antibody (1:50, Sigma-Aldrich). Scale bar: 50 µm. **E** DAPI-positive unrepaired cells over the whole cell population (tdTomato-positive), were quantified for at least 6 independent experiments. Experimental data together with mean (± SEM) are presented. Nonparametric Mann − Whitney test. **P value (< 0.01), ***P value (< 0.001), ****P value (< 0.0001). **F** Mean percentage of unrepaired cells represented in E are indicated in the second column. Mean percentage of “repaired cells over the whole cell population” (column 3) is calculated by subtracting the “unrepaired / whole cell population” rate to 13.6%, which corresponds to the rate of damaged and unrepaired cells in the absence of Ca^2+^ that prevents membrane resealing. “Repaired / damaged population” rate (column 4) is calculated as the percentage of repaired cells considering that the number of damaged cells is 13.6

To detect differences in membrane repair after shear stress, we stained cells with DAPI after damaging the plasma membrane by repeatedly forcing the cells through a 30 Gauge needle (Fig. 3A). To quantify differences in repair, we measured the ratio of DAPI to tdTomato fluorescence because DAPI only enters cells with damaged plasma membrane, whereas tdTomato labels all the cells. Cells are unable to repair their membrane in the absence of extracellular Ca^2+^, so cells damaged in this condition were used as a positive control. We observed 13.6 ± 2.3% of unrepaired cells after shear stress in the absence of Ca^2+^, a value much higher than in the absence of shear stress (0.7 ± 0.5%) (Fig. 3B, E, F). In the presence of Ca^2+^, the value dropped to 3.8 ± 1.9%, suggesting that about 70% control cells were able to repair membrane damage (Fig. 3B, E, F). Electron microscopy was used to study the morphology of MDA-MB-231 cells after treatment by shear stress. Unlike untreated cells (Fig. S6A), many cells subjected to shear stress in the presence of Ca^2+^ appeared to have lysed (Fig. S6B). This phenomenon was increased in the absence of Ca^2+^, suggesting it resulted from membrane damage that was not repaired (Fig. S6C). In line with this observation, focal membrane damage was frequently observed in cells treated in the presence of Ca^2+^ (Fig. S6D, red square). The absence of spilt cytoplasmic contents indicated that the cells successfully repaired the damage. A large bleb consistently protruded from the site of damage (Fig. S6D, red arrowheads), as previous observed in skeletal muscle cells [15]. These results suggest that shear stress creates membrane damage, whose repair requires Ca^2+^, as previously reported for most, if not all, mechanical damage to the plasma membrane [46]. We then examined the response to shear stress after silencing ANXA2 expression. In the presence of Ca^2+^, 11.3 ± 5.9% of shANXA2 MDA-MB-231 cells failed to repair their membranes following shear stress, similar to the value for control cells in the absence of Ca^2+^ (Fig. 3C, E, F). This defect in membrane repair was also observed in ANXA2-silenced AsPC-1 cells (Fig. S7, control vs shANXA2). Through electron microscopy, we observed that most shANXA2 MDA-MB-231 cells appeared to have lysed (Fig. S6E), as in the absence of Ca^2+^ (Fig. S6C). Taken together, these results strongly suggest that shear stress causes membrane damage whose repair requires ANXA2. Finally, we examined the ability of an anti-ANXA2 antibody to inhibit membrane repair. We hypothesized that the antibody would enter the cells through the disrupted membrane and inhibit membrane repair by neutralizing ANXA2. Control MDA-MB-231 cells were subjected to shear stress in the presence of both Ca^2+^ and monoclonal anti-ANXA2 antibody. We observed 8.7 ± 3.0% of unrepaired cells, significantly more than the value for control cells damaged in the presence of Ca^2+^, and inferred that the presence of anti-ANXA2 antibody leads to a defect in membrane repair after shear stress (Fig. 3D–F). The inhibitory effect of anti-ANXA2 antibody in membrane repair was confirmed in AsPC-1 cells (Fig. S7, control vs anti-ANXA2). We conclude that ANXA2 is a crucial component in response to shear stress and that anti-ANXA2 antibodies can inhibit membrane repair despite being administered outside the cell.

### ANXA2 deficiency impairs MDA-MB-231 and AsPC-1 cell invasion

**Fig. 4:**
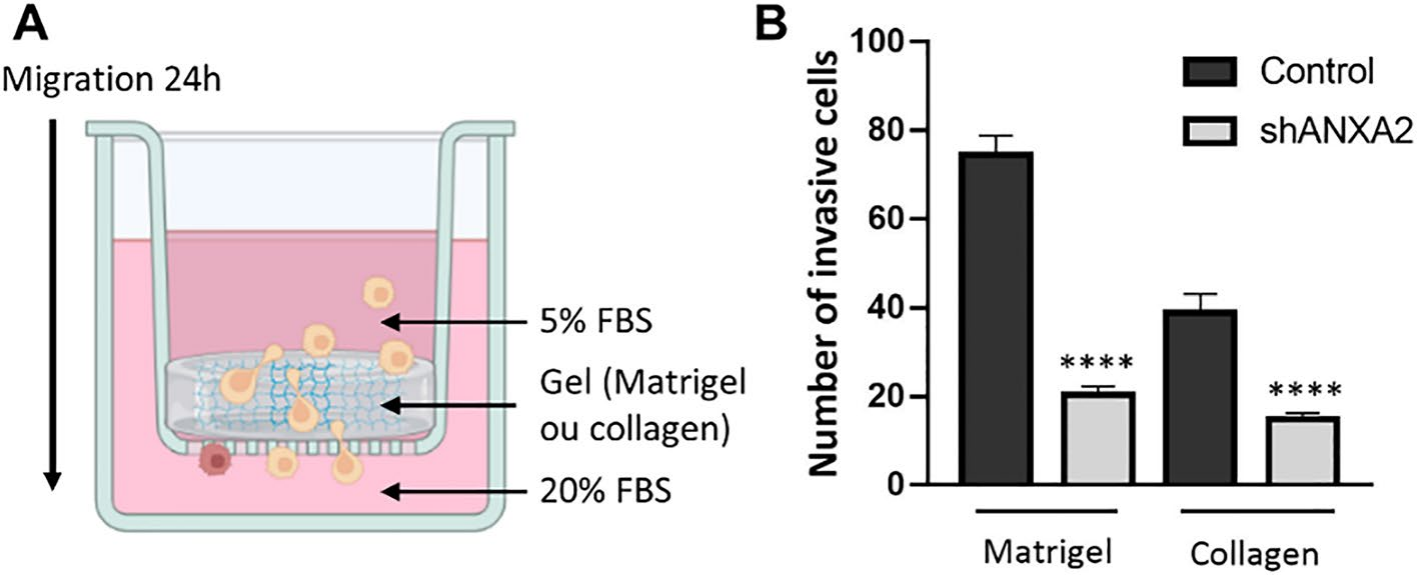
ANXA2 deficiency impairs MDA-MB-231 cell invasion. **A** Matrigel or dense collagen gel was produced into a transwell and cells were seeded in media with 5% FBS on top of the gel. As chemoattractant, 20% FBS-containing medium was added in the bottom of the well to allow cell invasion through the gel. Created using Biorender.com. **B** Number of control or shANXA2 MDA-MB-231 cells able to cross the gel was quantified. Unpaired Student’s *t* test. ****p < 0.0001

We next addressed the effect of ANXA2 silencing on MDA-MB-231 and AsPC-1 cell migration using a transwell invasion assay. We allowed cells to migrate through Matrigel or a dense collagen gel from medium containing 5% FBS in the upper chamber to medium containing 20% FBS in the lower chamber (Figs. 4A and S8). After 24 h of incubation, cells in the lower chamber were stained with crystal violet and counted. In the absence of ANXA2, the number of MDA-MB-231 cells in the lower chamber decreased by 72% and 62% for inserts covered with Matrigel and collagen, respectively (Fig. 4B). A similar result was observed with AsPC-1 cells with a decrease of 50% and 40% between control and shANXA2 cells for inserts covered with Matrigel and collagen, respectively (Fig. S9). We conclude that ANXA2 deficiency reduces the invasiveness of MDA-MB-231 and AsPC-1 cells.

### ANXA2 is required for metastasis in vivo in mice

**Fig. 5:**
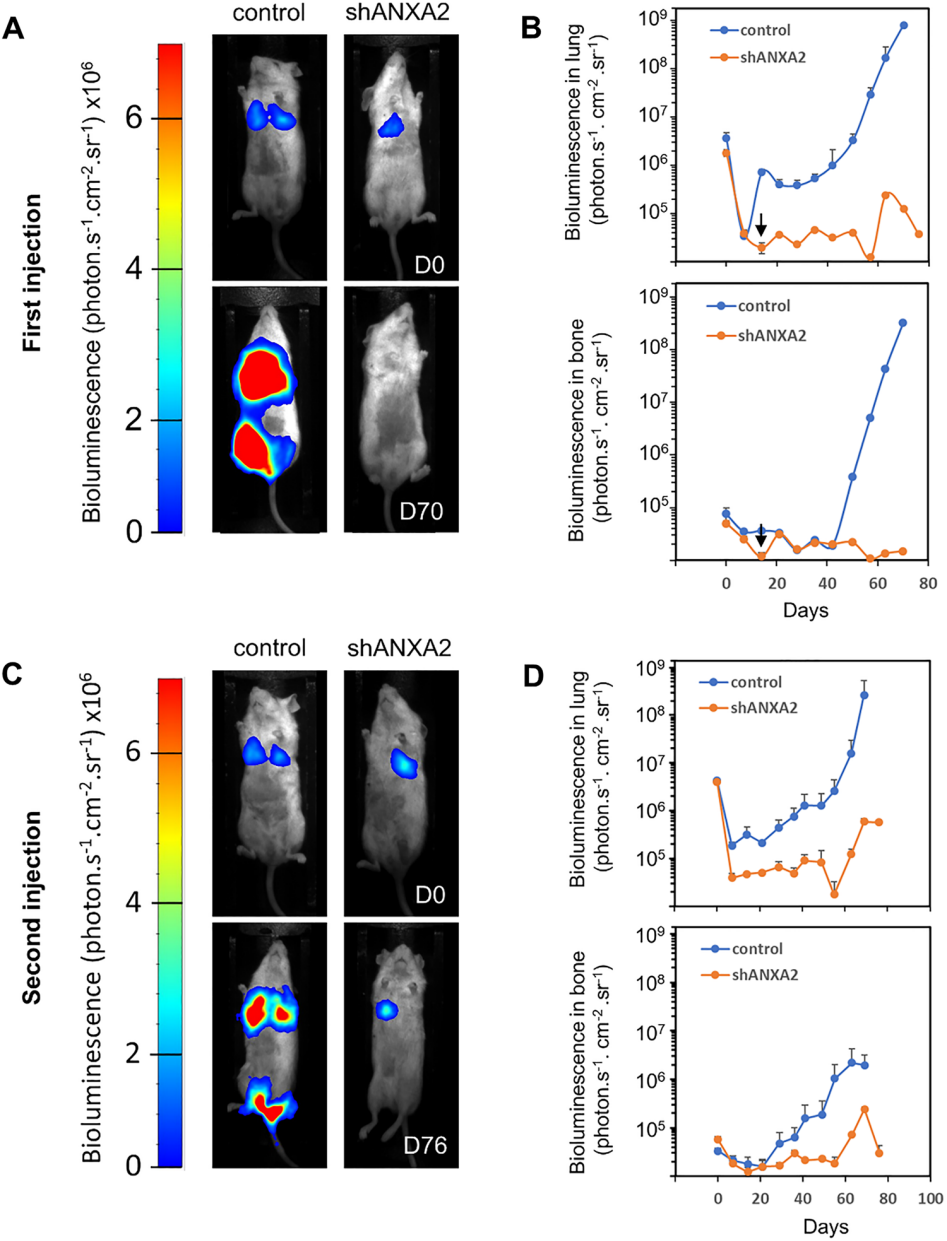
Genetic inhibition of ANXA2 prevents tissue colonization of MDA-MB-231 cells in NOD SCID mice. **A** Control or shANXA2 MDA-MB-231 cells were injected in NOD SCID mice as described in the legend of the Supplementary Fig. S10. Representative bioluminescence images of NOD SCID mice 30 min (D0) or 70 days (D70) after injection of MDA-MB-231 cells are presented. **B** Bioluminescence quantification in lungs and bone for control (*n* = 5) or shANXA2 (*n* = 5) MDA-MB-231 cells. The black arrow points the time when some mice (*n* = 4), initially injected with shANXA2 MDA-MB-231 cells and exhibiting low signal in bioluminescence imaging, were injected with control (*n* = 2) or ANXA2 shRNA (*n* = 2) MDA-MB-231 cells. ** C** Representative bioluminescence images of NOD SCID mice, initially injected with shANXA2 MDA-MB-231 cells and, injected a second time with either control or shANXA2 MDA-MB-231 cells, at 30 min (D0) or 76 days (D76) after the second injection. **D** Bioluminescence quantification in lungs and bone for control (*n* = 2) or shANXA2 (*n* = 2) MDA-MB-231 cells after the second injection

To investigate the role of ANXA2 in tumor progression, we labelled control and shANXA2 MDA-MB-231 cells with a luciferase reporter and injected the cells into the tail vein of NOD SCID mice (Figs. 5A, B and S10) and RAG mice (Fig. S11). The control cells engrafted in the lungs and bones, particularly in the hind legs (Figs. 5A,  and S12). The ANXA2-deficient cells were much less effective at colonizing the lungs and failed to engraft in the bones (Figs. 5A, B and S11). To confirm this result, some of NOD SCID mice initially injected with shANXA2 MDA-MB-231 cells, which did not show tumor development after the first injection (Fig. 5B, black arrow), were injected a second time with control or ANXA2-deficient MDA-MB-231 cells (Fig. 5C, D). Again, we observed that shANXA2 MDA-MB-231 cells formed fewer metastases in the lungs and bone as compared to control cells (Fig. 5C, D). We conclude that ANXA2 is required for metastasis in mice.

### ANXA2 is required for metastasis in vivo in zebrafish

**Fig. 6:**
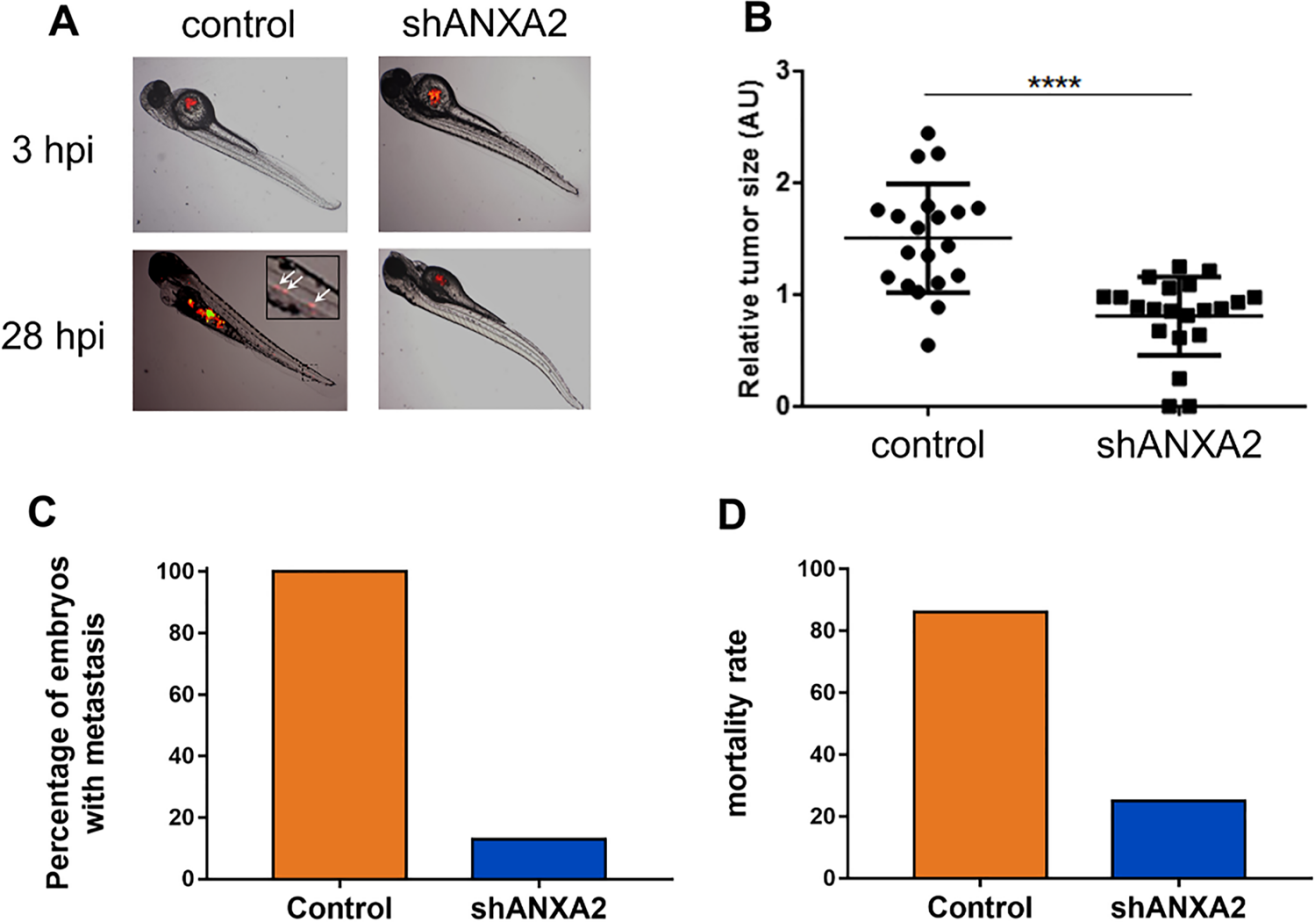
Genetic inhibition of ANXA2 prevents invasion and metastasis processes of MDA-MB-231 cells in zebrafish. **A** Control (*n* = 21) or shANXA2 MDA-MB-231 (*n* = 24) cells were injected in the perivitelline space of Casper zebrafish embryos. Tumor imaging was done by fluorescence microscopy at 3-, 28- and 52 hpi through the tdTomato fluorescent protein constitutively expressed in MDA-MB-231 cells. Inset displays magnified image of a portion of the tail with metastases (white arrows). **B** The relative tumor size within the perivitelline space has been measured in embryos either injected by control or shANXA2 cells. **C** The percentage of embryos, which presented caudal or head metastases was quantified at 28 hpi. **D** Mortality rate was calculated at 52 hpi

To confirm the lower propensity of ANXA2-deficient cells to metastasize, we injected control or shANXA2 MDA-MB-231 cells (Figs. 6A and S13) and control or shANXA2 AsPC-1 cells (Fig. S14) into the yolk sac of zebrafish larvae and quantified metastases formed in the tail and/or head of the fish. At 28 h post-injection (hpi), we observed that the relative growth of the tumor within the yolk sac was much lower after injection of shANXA2 MDA-MB-231 cells as compared to control cells, suggesting reduced survival of ANXA2-deficient cells (Fig. 6B). We also observed that all fish injected with the control MDA-MB-231 cells showed caudal and/or cranial metastases at 28 hpi, as compared to 13% for shANXA2 MDA-MB-231 cells injected fish (Fig. 6C). This reduced ability of shANXA2 cells to metastasize was confirmed with AsPC-1 cells (Fig. S14). Finally, we observed that the mortality rate was 86% for fish injected with control MDA-MB-231 cells but only 25% for those injected with ANXA2-deficient MDA-MB-231 cells at 52 hpi (Fig. 6D). We conclude that ANXA2 is required for engraftment and metastasis in zebrafish.

## Discussion

Involvement of annexins in cancer progression has already been reported [41, 42, 47, 48]. Nevertheless, given the multifunctionality of this protein family, which has been shown to participate in cell growth, proliferation, motility, and lipid/glucose homeostasis [47, 49], it is often difficult to decipher which is the main mechanism that may promote tumor invasion and metastasis. Here, we focused on the role played by annexins on membrane repair as a promoter of the cancer progression. The main conclusion from this study is that efficient repair of membrane damage is required for tumor invasion and metastasis. We hypothesized that cancer cells would require efficient membrane repair to cope with the physical stresses they are exposed to during invasion and metastasis. To test this assumption, we used two different animal models in which inhibition of ANXA2 gene expression and protein function drastically reduced the invasion and metastasis of breast and pancreatic cancer cells. Besides, we showed that membrane repair of these cancer cells is strictly dependent on the presence of ANXA2, whether the type of membrane damage either by laser ablation or shear stress. Among the most described membrane repair proteins are ANXA5 [4, 13, 14] and ANXA6 [5, 15, 50, 51]. We showed recently that ANXA5 and ANXA6 silencing prevents metastasis of breast cancer cells in vivo [41]. Even though ANXA2, ANXA5 and ANXA6 belong to the same family, they exhibit distinct roles in membrane repair and cannot compensate the absence of one or the other. ANXA2 acts within the cytoplasm to form the lipid patch, while ANXA5 and ANXA6 act at the plasma membrane, for strengthening the injured membrane and inducing membrane curvature, respectively [31, 52]. ANXA2 forms complexes with S100A10 and S100A11 [53] that participate in membrane repair [9, 12]. In addition, S100 proteins are associated with tumour metastasis as well as poor prognosis in various cancers [11, 23, 54]. Altogether, these data strongly reinforce the link between membrane repair and metastasis.

In skeletal muscle cells, ANXA2 participates in lipid patch formation where it is recruited to the damaged plasma membrane by interacting with dysferlin [3, 31]. The mechanism of membrane repair is best understood in muscle cells. Shearing forces are one of the major mechanical constraints applied to cancer cells during their dissemination [16] but little is known about membrane repair in cancer cells. Since ANXA2 interacts with actin, it could be also involved directly in cell migration or proliferation [55], but we show that these processes are largely unaffected in ANXA2-deficient MDA-MB-231 or AsPC-1 cells. Hence, we propose that tumor invasion and metastasis are affected by a defect in membrane repair.

ANXA2 is highly expressed in invasive tumor cells. We propose that this is an adaptation to survive the membrane damage these cells suffer during invasion and metastasis. Since normal cells are not exposed to these stresses, we speculate that inhibition of membrane repair will disproportionately affect invasive cancer cells. This means inhibitors of membrane repair should have a high therapeutic index. We show that an inhibitory antibody against ANXA2 is able to inhibit repair when administered in the culture medium. To affect repair the antibody must enter the cell, and we propose that it does so at sites of membrane damage. Intriguingly, this means therapeutic antibodies should only affect cells with membrane damage, potentially further increasing the therapeutic index. This result confirms a previous report on ovarian cancer cells showing that an anti-ANXA2 antibody significantly decreases invasion of the chick embryo chorioallantoic membrane and inhibits tumor growth and metastasis in nude mice [56]. It opens new routes using immunotherapy to tackle cancer cell dissemination. An alternative to antibody therapy would be to use small molecule inhibitors or peptides. Drugs used to treat psychiatric and allergic conditions, such as trifluoperazine, have been reported to inhibit annexin-mediated membrane repair [57], and a synthetic cell-penetrating peptide that interacts with ANXA2 has been shown to reduce metastasis to murine lungs [58]. Major compressive and shearing forces are encountered during invasion and metastasis. We conclude that repair of membrane damage is a new target for cancer therapy that could be used to prevent invasion and metastasis of poor prognosis tumors.

### Supplementary Information

Below is the link to the electronic supplementary material.

Supplementary file1 (DOCX 9829 KB)

## Data Availability

Note applicable.

## Abbreviations

ANXA1: Annexin-A1
ANXA2: Annexin-A2
ANXA5: Annexin-A5
ANXA6: Annexin-A6
Ca^2+^: Calcium
DPBS: Dulbecco’s phosphate buffer saline
MOI: Multiplicity of infection
