# Supplementary material for: Inhibition of the membrane repair protein annexin-A2 prevents tumor invasion and metastasis
Source: Cell Mol Life Sci. 2023 Dec 13;81(1):7. doi: 10.1007/s00018-023-05049-3 (PMC10719157; doi:10.1007/s00018-023-05049-3)
Supplement: Supplementary file 1 — Supplementary file1 (DOCX 9829 KB) [file 18_2023_5049_MOESM1_ESM.docx]

**SUPPLEMENTARY MATERIAL FOR:**

**Inhibition of the membrane repair protein annexin-A2 prevents tumor invasion and metastasis**

Gounou C.^1^, Rouyer L.^2^, Siegfried G.^2,6^, Harté E.^3^, Bouvet F.^1^, d’Agata L.^1^, Darbo E. ^2^, Lefeuvre M.^1^, Derieppe M.A.^4^, Bouton L.^2^, Mélane M.^3^, Chapeau D.^1^, Martineau J.^4^, Prouzet-Mauleon V.^2,5^, Tan S.^1^, Souleyreau W.^2^, Saltel F.^2^, Argoul F.^3^, Khatib A.M.^2,6,7^, Brisson A.R.^1^, Iggo R.^2^, Bouter A.^1,*^

^1^ Univ. Bordeaux, CNRS, Bordeaux INP, CBMN, UMR 5248, F-33600 Pessac, France

^2^ Univ. Bordeaux, INSERM, BRIC, U 1312, F-33000 Bordeaux, France

^3^ Univ. Bordeaux, CNRS, LOMA, UMR 5798, F-33400 Talence, France

^4^ Univ. Bordeaux, Animalerie Mutualisée, Service Commun des Animaleries, F-33000 Bordeaux, France

^5^ CRISPRedit, TBMcore, UAR CNRS 3427, Inserm US 005, Univ. Bordeaux, France

^6^ XenoFish, B2 Ouest, Allée Geoffroy St Hilaire CS50023, Pessac 33615, France

^7^ Bergonié Institute, Bordeaux, France

*Corresponding author

Mailing address: Bât. B14, Allée Geoffroy Saint Hilaire, 33600 Pessac, France

E-mail: anthony.bouter@u-bordeaux.fr; Tel: +33 540006860

**
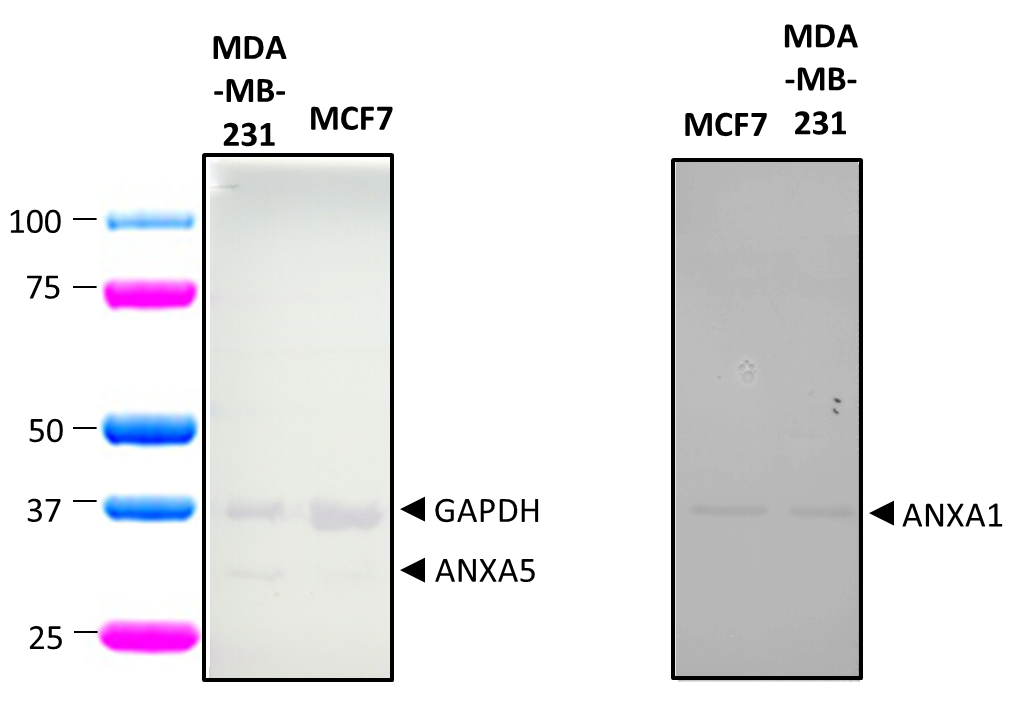
**

**Supplementary Figure S1.** Invasiveness properties of breast cancer cells are correlated with high expression of ANXA1 and ANXA2. Representative image of Western blotting showing the revelation of ANXA1 and ANXA5 (not analyzed, out of the scope for this article) in MDA-MB-231 and MCF7 cells, compared to GAPDH (loading control). Apparent molecular weights of the prestained protein marker are indicated on the left (in kDa). The histogram displaying mean values (± SEM) of the ratio ANXA1/GAPDH from five independent experiments, is presented in Figure 1B.

**
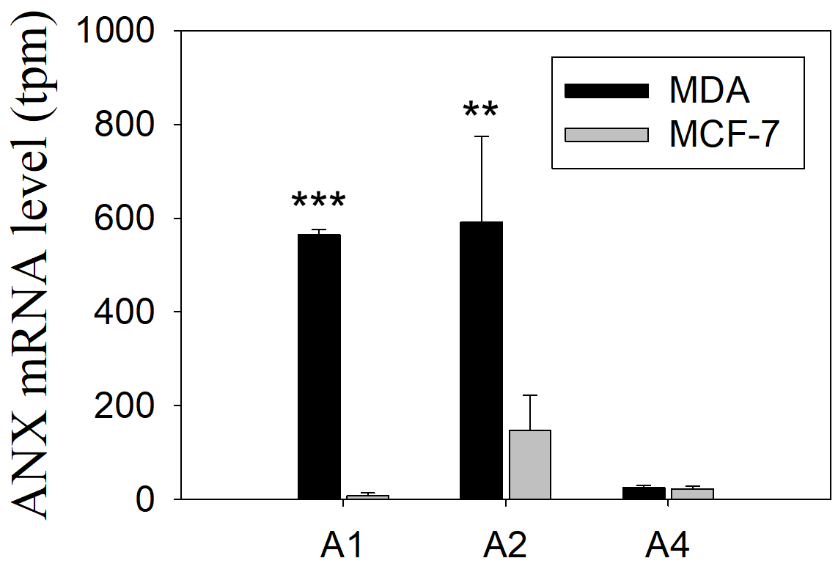
**

**Supplementary Figure S2.** Comparison of ANX mRNA expression between MDA-MB-231 and MCF-7 cells. Analysis was performed using the EMBL-EBI expression Atlas repository (<https://www.ebi.ac.uk/gxa/home).> Two and seven independent experiments were reported for MDA-MB-231 and MCF7 cells, respectively [1–5]. Non-parametric Mann-Whitney test. ***: p < 0.001, **: p < 0.005. Tpm means transcripts per million.

**
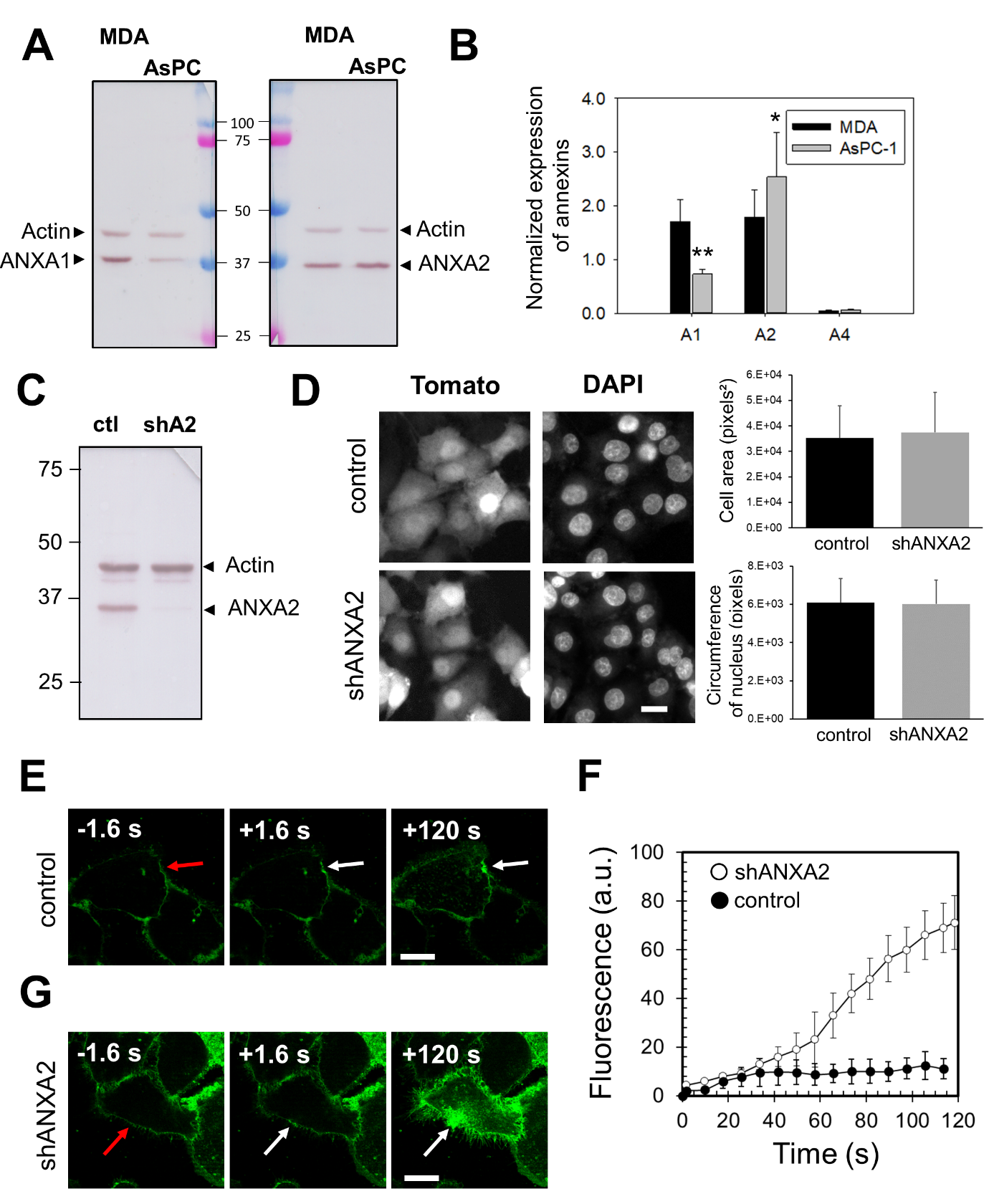
Supplementary Figure S3.** Highly expressed in AsPC-1 cells, ANXA2 promotes membrane repair. **A** Representative image of western-blot analysis showing the revelation of ANXA2 in AsPC-1 compared to MDA-MB-231. Actin was used as a loading control. **B** The histogram presents mean values (± SEM) of the ratio ANX/Actin from five independent experiments, analyzed by the gel analysis plugging of ImageJ. Student t-test for independent samples. *: p < 0.05. **: p < 0.01. **C** ANXA2 deficient AsPC-1 cells were generated by shRNA transduction strategy. The cellular content of ANXA2 in AsPC-1 cells transduced with lentiviral particles containing shRNA targeting ANXA2 (shA2) or a scrambled shRNA (ctl) was quantified by Western blotting. **D** Control and shANXA2 AsPC1 cells were imaged by fluorescence microscopy. Right-hand histograms display mean cell area (in pixels²) and nuclei circumference (in pixels) measured by the imageJ software using Tomato and DAPI images. The mean values (+/−SEM) were calculated from at least 30 cells from three independent experiments. No statistical difference (student t-test) was observed for the two parameters. Scale bar: 10 µm. **E, G** Sequences of representative images showing the response of a control (E) or shANXA2 (G) AsPC-1 cell to a membrane damage performed by 110-mW infrared laser irradiation, in the presence of FM1-43 (green). In all figures, the area of membrane irradiation is marked with a red arrow before irradiation and a white arrow after irradiation. Scale bars: 10 μm. **F** Kinetic data represent the FM1−43 fluorescence intensity for control (black filled circles) or shANXA2 (empty circles) AsPC-1 cells, integrated over whole cell sections, averaged for about 30 cells (+/−SEM).

**
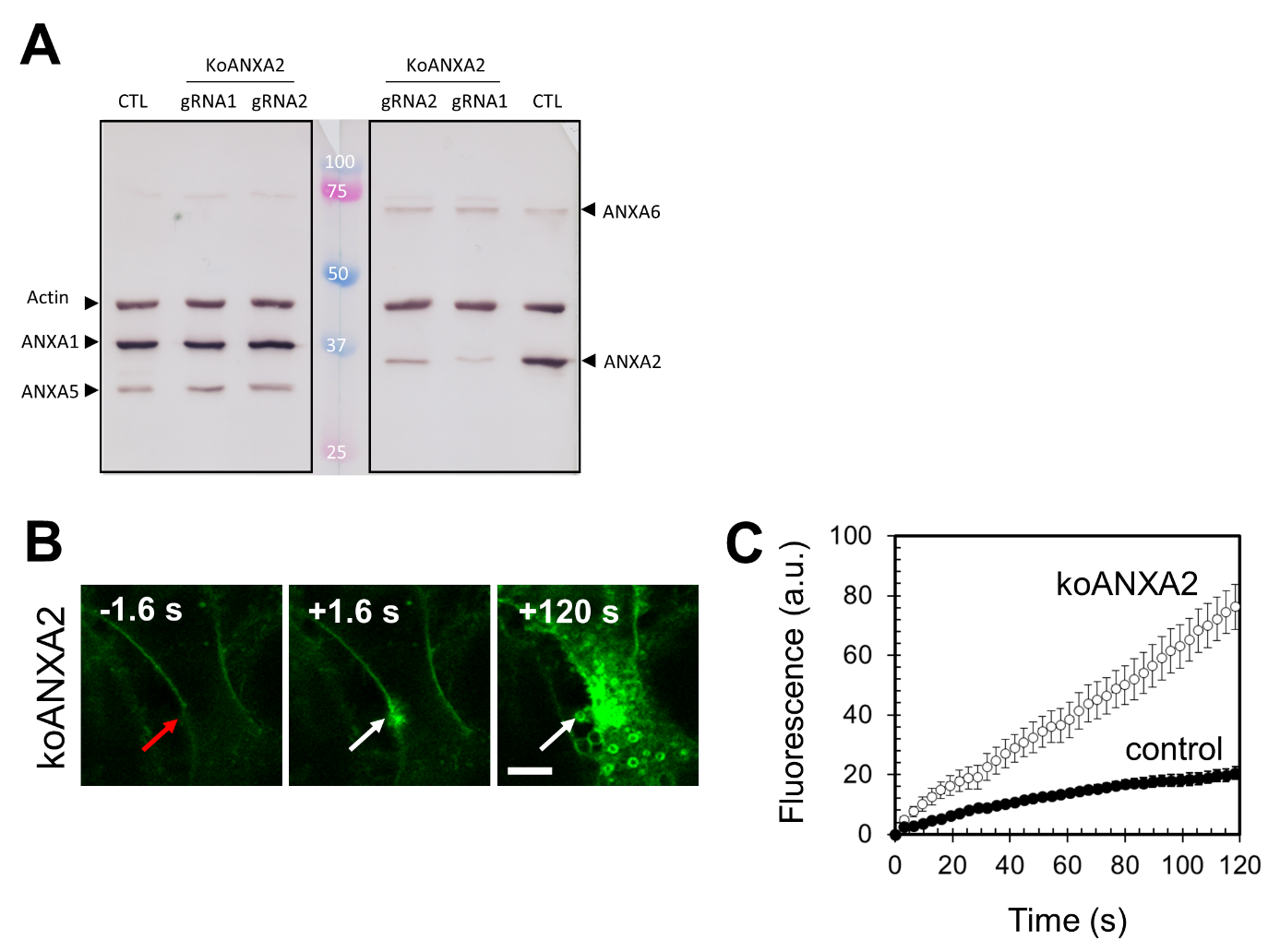
Supplementary Figure S4.** Response of koANXA2 MDA-MB-231 cells to laser ablation. ANXA2-knock-out MDA-MB-231 cells were generated by CRISPR-Cas9 approach, using either gRNA1 or gRNA2. **A** The specific extinction of the ANXA2 expression in both was confirmed by Western-blotting, since ANXA1, A5 and A6 expression was not affected. Apparent molecular weights of the prestained protein marker are indicated (in kDa). **B** Sequences of representative images (n = 30) showing the response of a koANXA2 MDA-MB-231 cell (gRNA1) to a membrane damage performed by 110-mW infrared laser irradiation, in the presence of FM1-43 (green). The area of membrane irradiation is marked with a red arrow before irradiation and a white arrow after irradiation. Scale bar: 10 μm. **C** Kinetic data represent the FM1−43 fluorescence intensity integrated over whole cell sections, averaged for about 30 cells (+/−SEM). For a majority of control MDA-MB-231 cells, the fluorescence intensity reached a plateau after about 80 s (black filled circles). For koANXA2 MDA-MB-231 cells, a continuous and large increase of the fluorescence intensity was observed (empty circles), indicating the absence of membrane resealing.


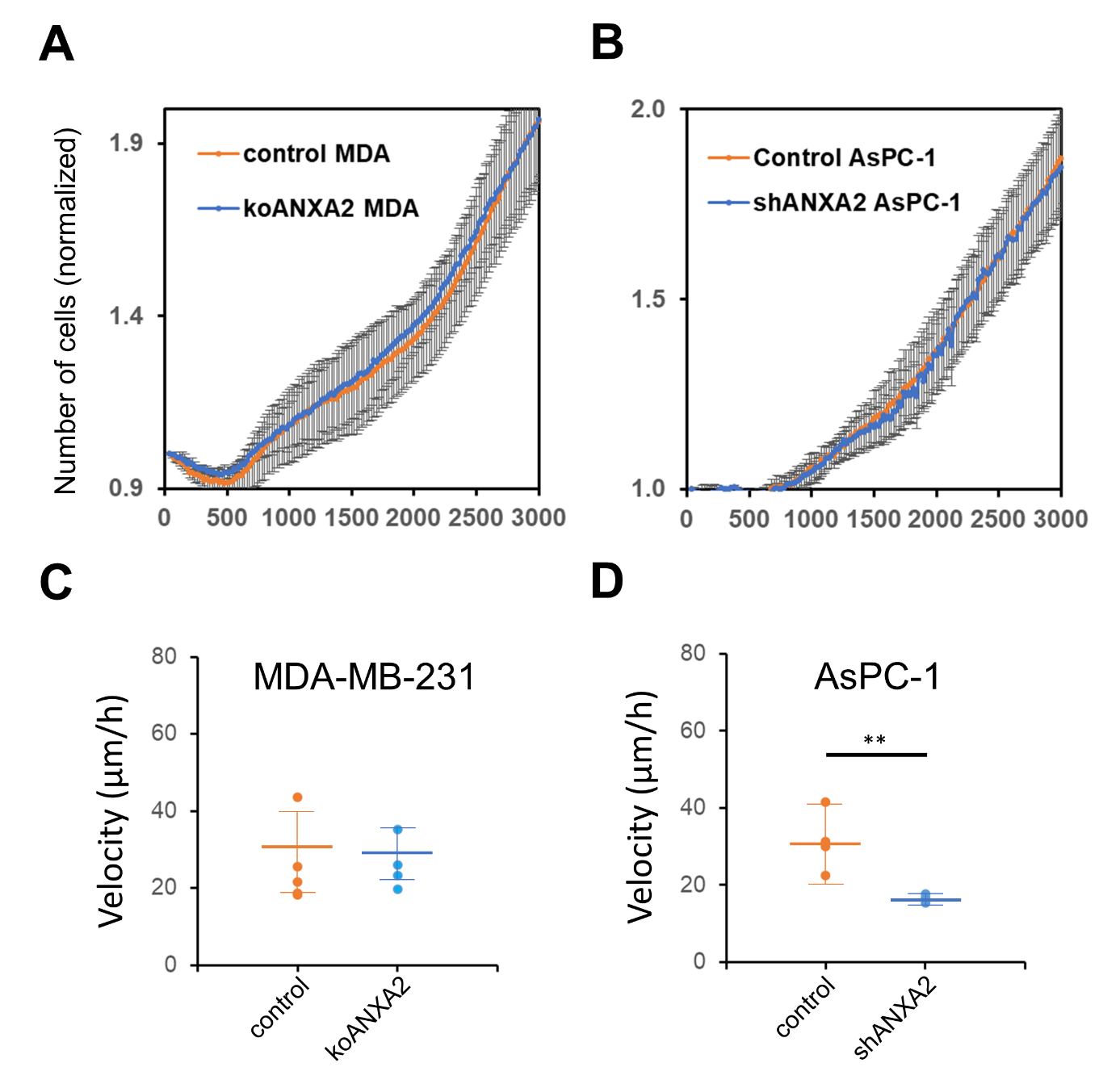


**Supplementary Figure S5.** Proliferation and 2D-migration studies of control ANXA2-deficient MDA-MB-231 and AsPC-1cells. Proliferation of control or koANXA2 MDA-MB-231 cells (A) and control or shANXA2 AsPC-1 cells (B) was monitored as described in the legend of Figure 2A-B. 2D-migration of control or koANXA2 MDA-MB-231 cells (C) and control or shANXA2 AsPC-1 cells (D) was analyzed as described in the legend of Figure 2C-D.


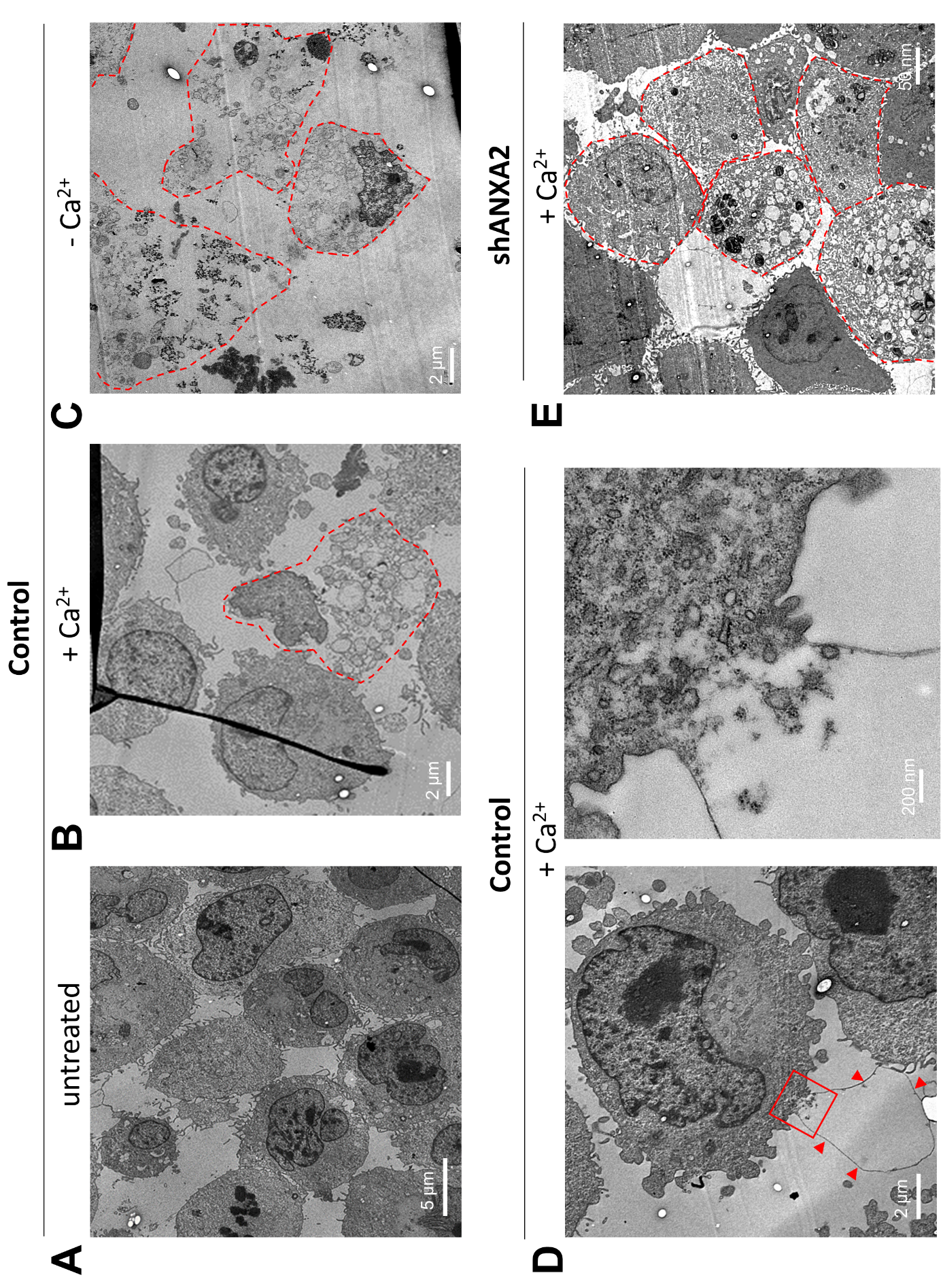


**Supplementary Figure S6.** Control and shANXA2 MDA-MB-231 cells submitted to the shear-stress treatment analyzed by TEM. Control cells submitted (B-D) or not (A) to the shear-stress treatment were fixed, embedded in Epon-Araldite and ultra-thin sections imaged by TEM. Shear-stress treatment was performed with (B, D and E) or without (C) 2 mM Ca^2+^. **A** In the absence of treatment, the cells appear with an electron-dense cytoplasmic content and a conspicuous cell membrane. **B** The shear-stress treatment led frequently to the observation of cells stripped of their content (red dotted line). **C** In the absence of Ca^2+^, this effect was accentuated (cells with red dotted line). **D** In the presence of Ca^2+^, many cells exhibited isolated micrometric membrane damage (red square), which seemed to be resealed. The right-hand image displays a high-magnification of the area present within the red square. Red arrowheads point out the large bleb protruded from the site of damage. **E** Most shANXA2 MDA-MB-231 cells submitted to the shear-stress treatment appeared to have lysed (red dotted line) in the presence of Ca^2+^.


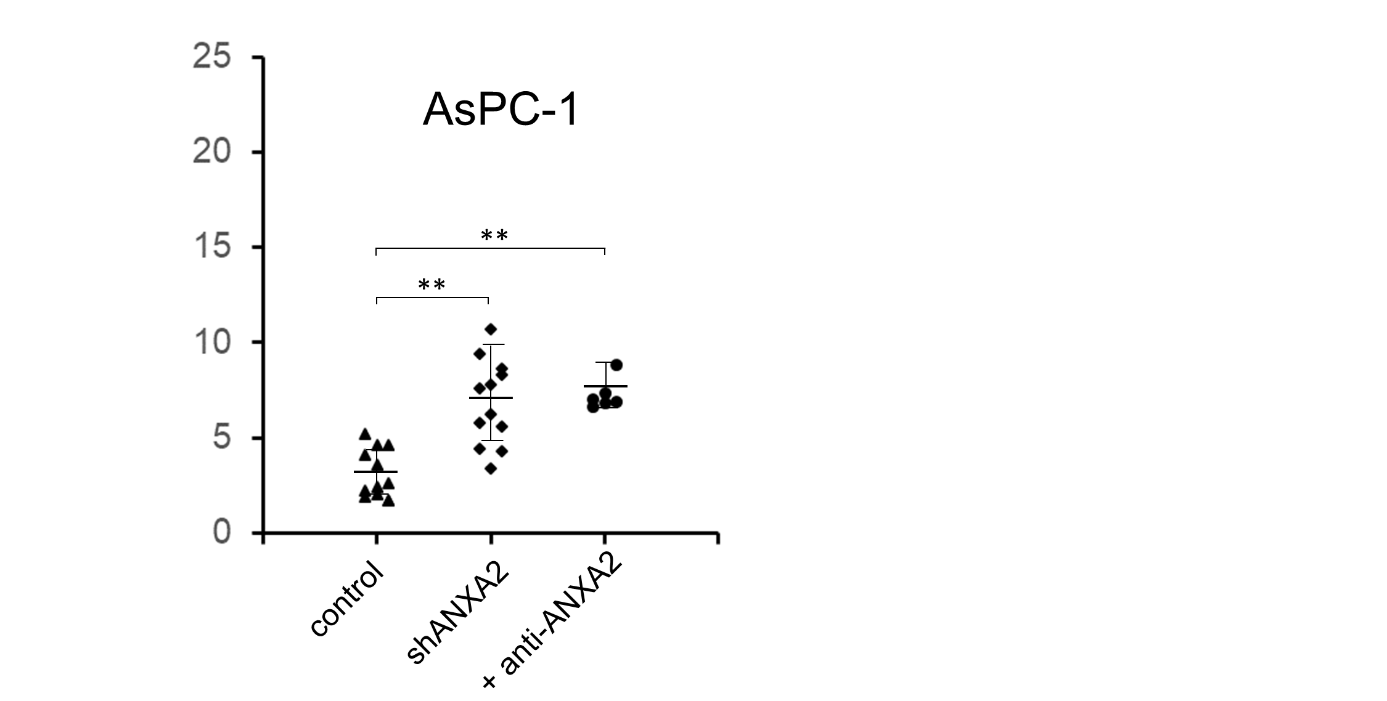


**Supplementary Figure S7.** Response of control or shANXA2 AsPC-1 cells to membrane injury by shear-stress treatment. The experiment was performed as described in the legend of Figure 3.


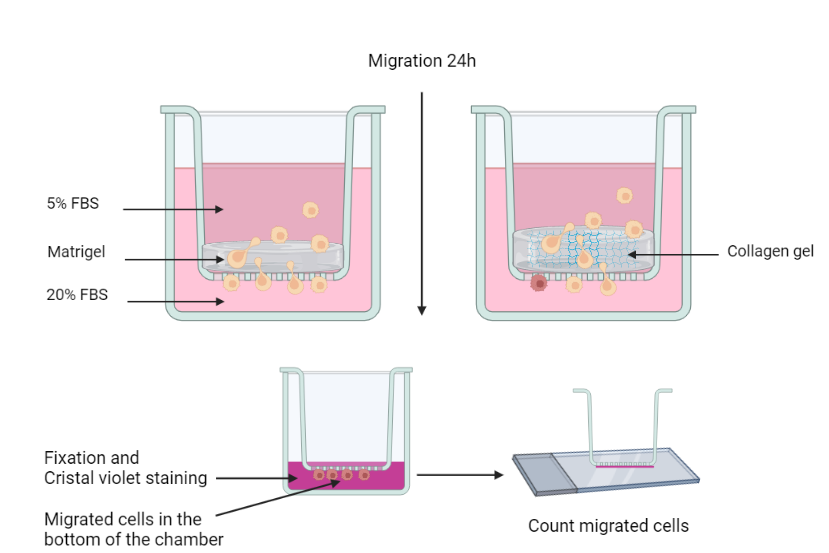


**Supplementary Figure S8.** Principle of 3D-invasion assay. Transwell chamber (8.0 µm pore size) was coated with Matrigel or fibrillar collagen in a 24-well plate. Control or shANXA2 cells were seeded in complete medium with 5% of FBS and the chamber was immerged in 20% FBS complete medium, used as chemoattractant. After 24 h, the media was carefully removed, cells were fixed with 4% PFA and stained with crystal violet. A phase-contrast microscope was used to image migrated cells in the lower chambers. Created using [Biorender.com](http://biorender.com).


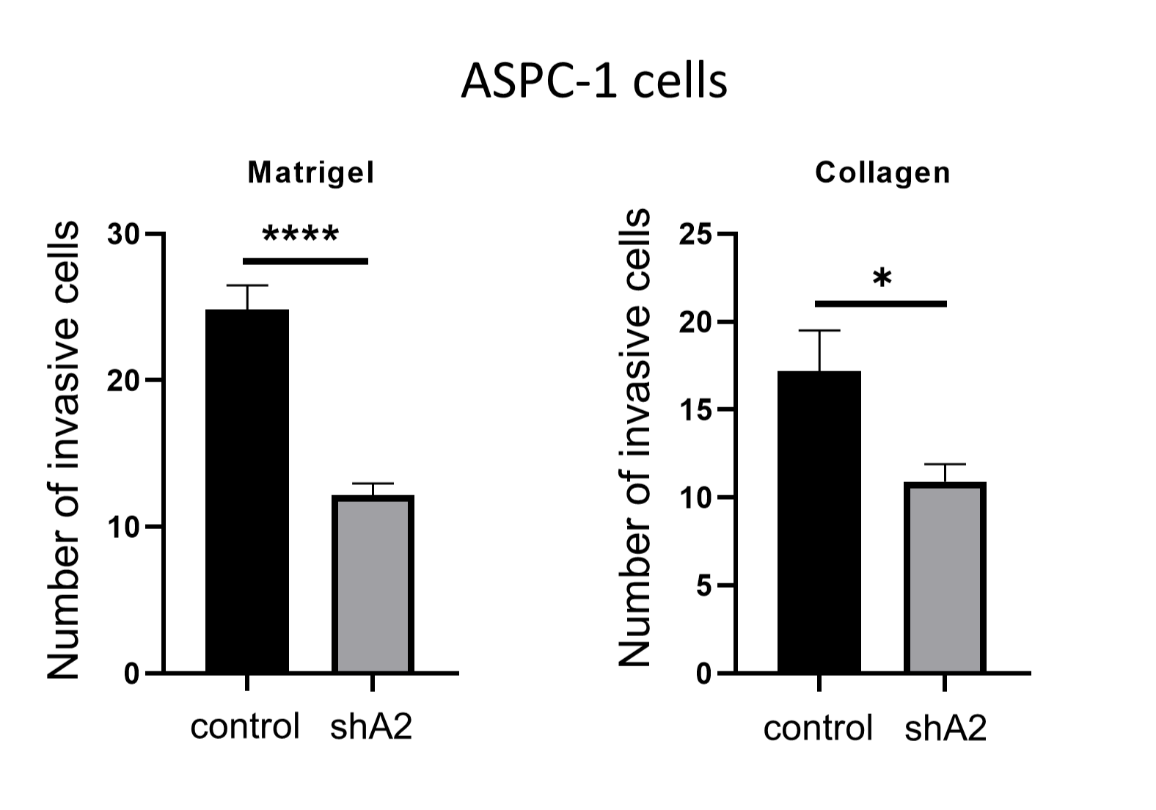


**Supplementary Figure S9.** ANXA2 deficiency impairs AsPC-1 cell invasion. Number of control or shANXA2 (shA2) AsPC-1 cells able to cross the gel was quantified, as described in the legend of Figure 4 and Supplementary Figure S8. Unpaired Student t-test. ****: p < 0.0001.

**
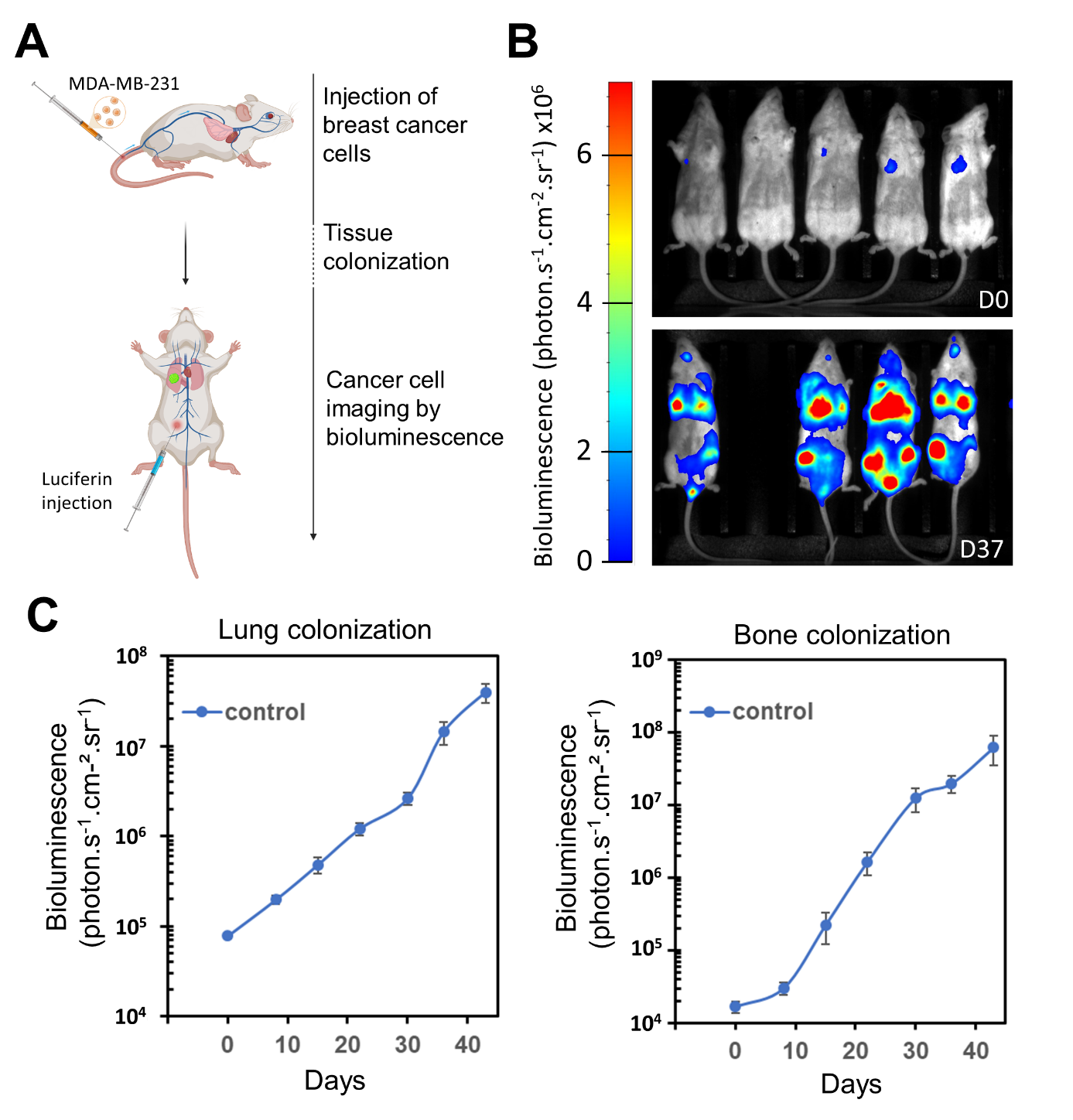
Supplementary Figure S10.** Murine lung and bone colonization by MDA-MB-231 cells in mice. **A** Scheme of the experimental procedure. MDA-MB-231 cells were injected into the tail vein of mice and 30 min after cell injection, and then every week, *in vivo* cell tracking was performed by intraperitoneal injection of luciferin enabling bioluminescence imaging. Created using [Biorender.com](http://biorender.com). **B** Representative bioluminescence images of RAG mice 30 min (D0) or 37 days (D37) after injection of control MDA-MB-231 cells. **C** Bioluminescence quantification in murine lungs and bone averaged for 10 mice (+/−SEM).


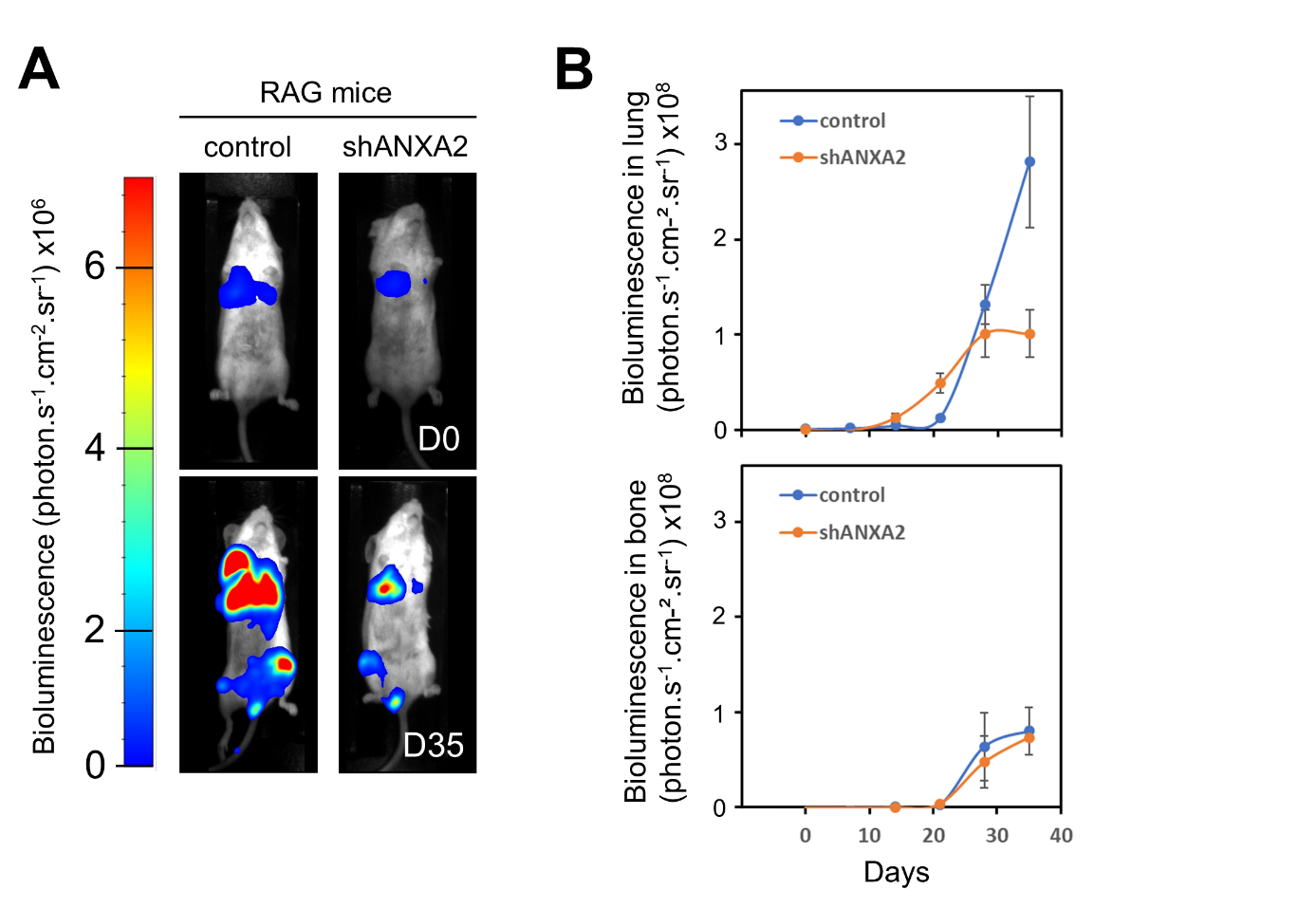


**Supplementary Figure S11.** Genetic inhibition of ANXA2 prevents lung colonization of MDA-MB-231 cells in RAG mice. **A** Control or shANXA2 MDA-MB-231 cells were injected in RAG mice as described in the legend of the Supplementary Fig. S10. Representative bioluminescence images of RAG mice 30 min (D0) or 35 days (D35) after injection of MDA-MB-231 cells are presented. **B** Bioluminescence quantification in lungs and bone for control (n = 10) or shANXA2 (n = 10) MDA-MB-231 cells.


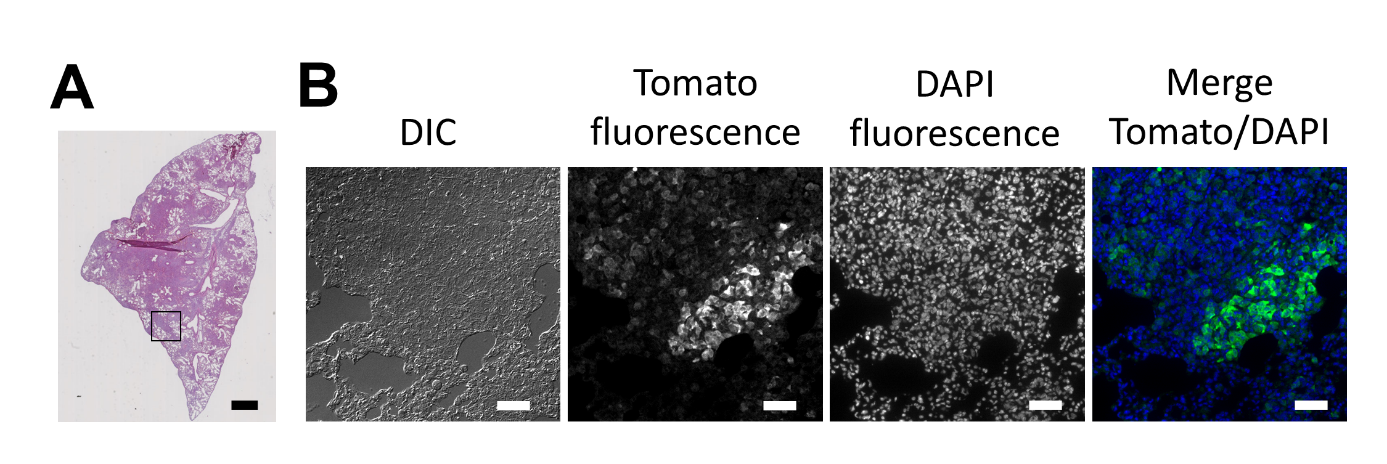


**Supplementary Figure S12.** Histological analysis of lungs from mice injected with control MDA-MB-231 cells. Lungs from mice injected with control cells were collected after mice euthanasia and fixed in PFA4%. **A** Representative image of a section labeled by hematoxylin-eosin and observed by Nanozoomer (Hamamatsu). Black square marks the area observed by immunocytofluorescence in successive sections (see B). Scale bar: 1 mm. **B** Representative images of a section immunostained for the tdTomato protein (green) and counterstained with DAPI (blue). Section was observed by differential interference contrast (DIC) and fluorescence microscopy. The immunostaining of the tdTomato protein reveals the presence of MDA-MB-231 cells within the murine lungs. Scale bar: 50µm.


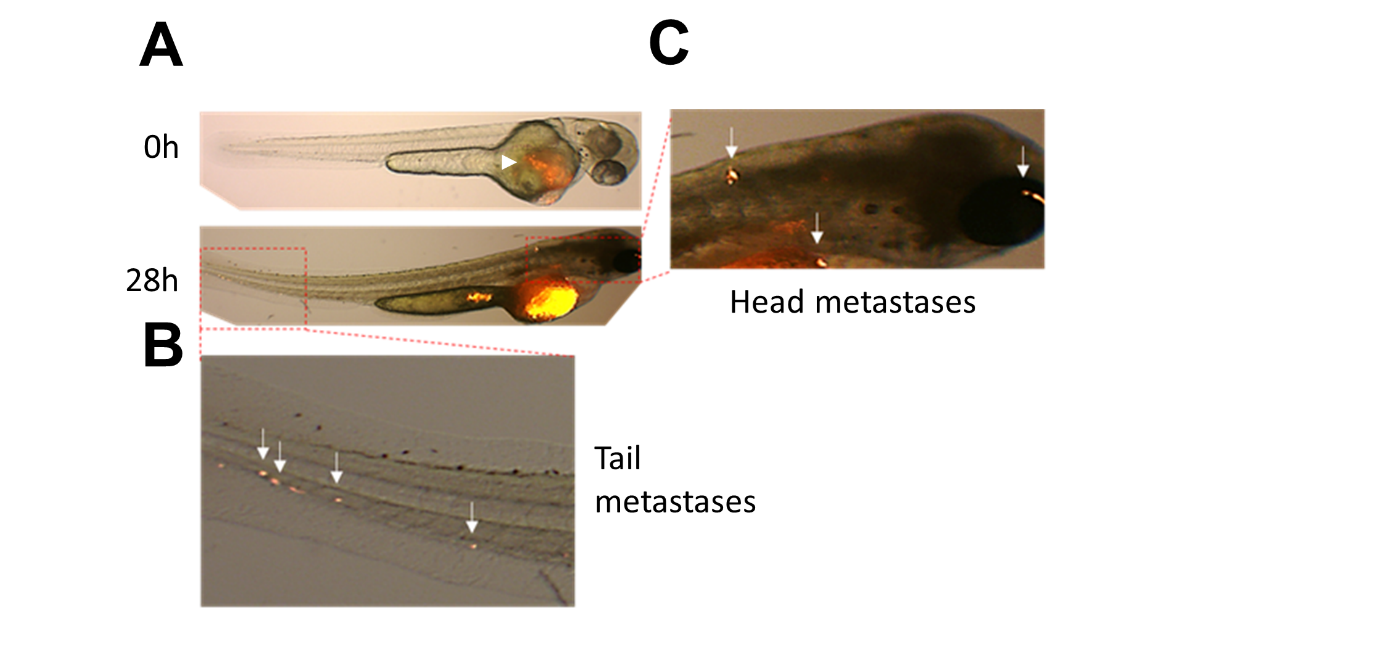


**Supplementary Figure S13.** Principle of metastasis analysis in zebrafish. tdTomato (red) expressing MDA-MB-231 or AsPC-1 cells were injected into the yolk sac of zebrafish larvae (A, white arrowhead) and metastases formed in tail (B, white arrows) and/or head of fish (C, white arrows) were quantified at 28 hpi by fluorescence microscopy imaging.


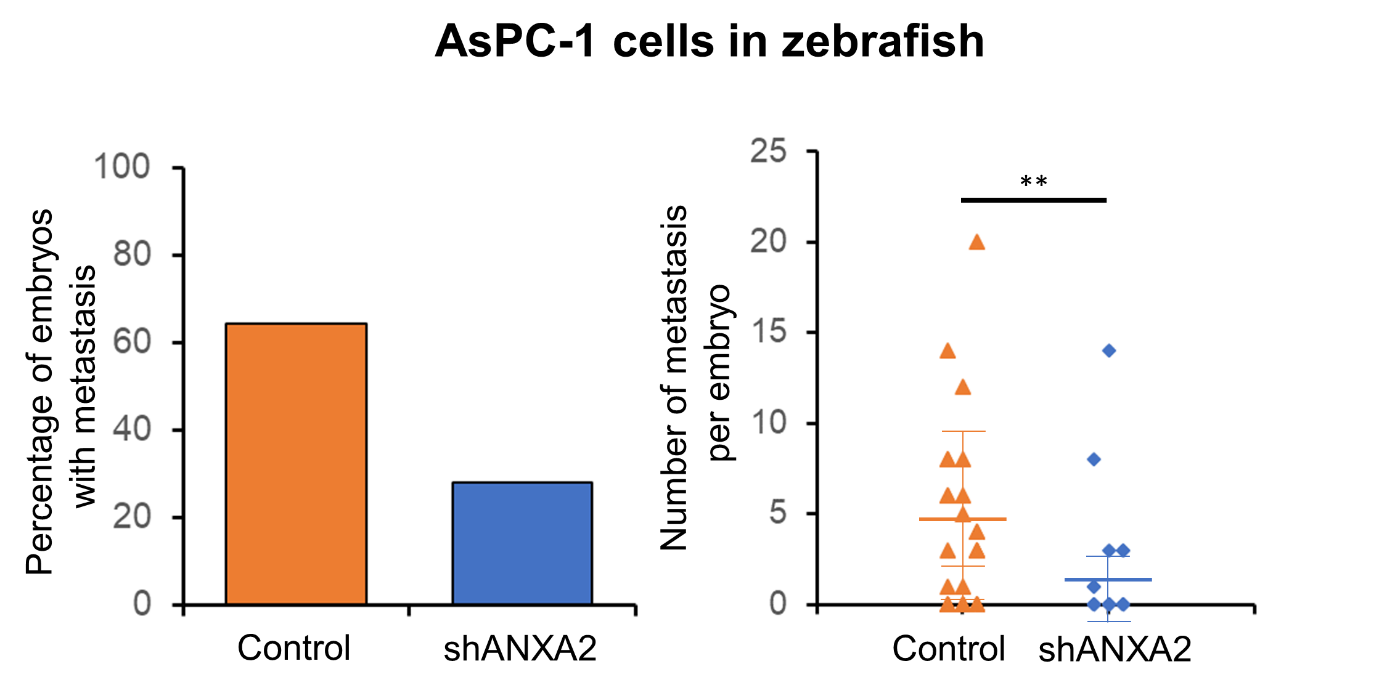


**Supplementary Figure S14**. Genetic inhibition of ANXA2 prevents metastasis of AsPC-1 cells in zebrafish. Control (n = 28) or shANXA2 AsPC-1 (n = 28) cells were injected in the perivitelline space of Casper zebrafish embryos, as described in the legend of Figure 6. The percentage of embryos, which presented caudal or head metastases, was quantified and the number of metastasis per embryo as well. Unilateral Student t-test, ** p-value < 0.01.

**References in Supplementary Material**

1. Jastrzebski K, Thijssen B, Kluin RJC, et al (2018) Integrative Modeling Identifies Key Determinants of Inhibitor Sensitivity in Breast Cancer Cell Lines. Cancer Res 78:4396–4410. https://doi.org/10.1158/0008-5472.CAN-17-2698

2. Barretina J, Caponigro G, Stransky N, et al (2012) The Cancer Cell Line Encyclopedia enables predictive modelling of anticancer drug sensitivity. Nature 483:603–607. https://doi.org/10.1038/NATURE11003

3. Ghandi M, Huang FW, Jané-Valbuena J, et al (2019) Next-generation characterization of the Cancer Cell Line Encyclopedia. Nature 569:503–508. https://doi.org/10.1038/S41586-019-1186-3

4. Deng X, Hiatt JB, Nguyen DK, et al (2011) Evidence for compensatory upregulation of expressed X-linked genes in mammals, Caenorhabditis elegans and Drosophila melanogaster. Nat Genet 43:1179–1185. https://doi.org/10.1038/NG.948

5. Djebali S, Davis CA, Merkel A, et al (2012) Landscape of transcription in human cells. Nature 489:101–108. https://doi.org/10.1038/NATURE11233
